# Tobacco drought stress responses reveal new targets for Solanaceae crop improvement

**DOI:** 10.1186/s12864-015-1575-4

**Published:** 2015-06-30

**Authors:** Roel C Rabara, Prateek Tripathi, R Neil Reese, Deena L Rushton, Danny Alexander, Michael P Timko, Qingxi J Shen, Paul J Rushton

**Affiliations:** Texas A&M AgriLife Research and Extension Center, Dallas, Texas 75252 USA; Molecular and Computational Biology Section, Dana & David Dornsife College of Letters, Arts and Sciences, University of Southern California, Los Angeles, CA USA; Department of Biology and Microbiology, South Dakota State University, Brookings, SD 57007 USA; Department of Biological Sciences, University of North Texas, Denton, TX 76203 USA; Metabolon, Inc., 617 Davis Drive, Durham, NC 277133 USA; Department of Biology, University of Virginia, Charlottesville, Virginia 22904 USA; School of Life Sciences, University of Nevada, Las Vegas, 89154 USA

## Abstract

**Background:**

The Solanaceae are an economically important family of plants that include tobacco (*Nicotiana tabacum* L.), tomato, and potato. Drought is a major cause of crop losses.

**Results:**

We have identified major changes in physiology, metabolites, mRNA levels, and promoter activities during the tobacco response to drought. We have classified these as potential components of core responses that may be common to many plant species or responses that may be family/species-specific features of the drought stress response in tobacco or the Solanaceae. In tobacco the largest increase in any metabolite was a striking 70-fold increase in 4-hydroxy-2-oxoglutaric acid (KHG) in roots that appears to be tobacco/Solanaceae specific. KHG is poorly characterized in plants but is broken down to pyruvate and glyoxylate after the *E. coli* SOS response to facilitate the resumption of respiration. A similar process in tobacco would represent a mechanism to restart respiration upon water availability after drought. At the mRNA level, transcription factor gene induction by drought also showed both core and species/family specific responses. Many Group IX Subgroup 3 AP2/ERF transcription factors in tobacco appear to play roles in nicotine biosynthesis as a response to herbivory, whereas their counterparts in legume species appear to play roles in drought responses. We observed apparent Solanaceae-specific drought induction of several Group IId WRKY genes. One of these, *NtWRKY69*, showed ABA-independent drought stress-inducible promoter activity that moved into the leaf through the vascular tissue and then eventually into the surrounding leaf cells.

**Conclusions:**

We propose components of a core metabolic response to drought stress in plants and also show that some major responses to drought stress at the metabolome and transcriptome levels are family specific. We therefore propose that the observed family-specific changes in metabolism are regulated, at least in part, by family-specific changes in transcription factor activity. We also present a list of potential targets for the improvement of Solanaceae drought responses.

**Electronic supplementary material:**

The online version of this article (doi:10.1186/s12864-015-1575-4) contains supplementary material, which is available to authorized users.

## Background

Among the flowering plants, the Solanaceae (nightshade family) ranks third (after grasses and legumes) as the most important crop for human beings [50]. This highly diverse group is comprised of 90 genera and 3000–4000 species, in which half of the species belongs to the genus Solanum [34]. Several members of this genus, such as tomato (*S. lycopersicum*), potato (*S. tuberosum*) and eggplant (*S. melongena*) are important to human diet. Other members of the family are utilized for drug production [34]. This angiosperm family is also interesting because a number of its members such as *Nicotiana* spp, *Solanum* spp., *Petunia* spp., and *Datura* spp are used as biological model systems [56].

Tobacco is an agriculturally important Solanaceae crop and is one of the most studied plants as biological model system [51]. It is a convenient plant for research because it can be easily transformed and has a relatively short generation time. A cell line (BY-2) is a popular system for functional genomics research because of its fast growth, responsiveness to a variety of plant hormones and ease of transformation. There is also significant interest in understanding genome evolution in tobacco and the Solanaceae [60].

Drought is one of the major constraints in crop production and affects 64% of the global land area [11,48,65]. It is the most common cause of severe food shortage in developing countries [10]. Using candidate gene approaches, there have been a number of reports of genes that improve drought tolerance in tobacco [35]. These include DREB and WRKY transcription factors, genes that alter the levels of trehalose and mannitol, and LEA genes [31,46,63,64,69,70]. The majority of these genes came from bacteria or other plant species and tobacco was used as a model system [31,46,63,64,69,70]. In order to improve drought responses in tobacco and other Solanaceae species, it is desirable to understand how these plants respond to drought stress at multiple levels in the plant. This allows a better understanding of primary and secondary metabolism and the interplay between transcriptional, posttranscriptional, translational and posttranslational regulation. However, there are few reports on metabolic changes in tobacco during drought stress and systems biology data that combine results from multiple different levels in the same samples appear to be lacking.

Here we present extensive data sets on the response of tobacco to drought stress at the physiological, mRNA, metabolite, and promoter levels. We identify novel aspects to the response, such as the accumulation of 4-hydroxy-2-oxoglutaric acid (KHG) in roots, and identify genes, metabolites, transcription factors, and promoters that are potential components of novel strategies to improve drought stress responses in Solanaceae crops. These data provide a framework for crop improvement and are timely because of the recent publication of the tobacco genome sequence [60]. They also provide extensive novel resources for comparative analyses.

## Results

### The physiological level

**Figure 1:**
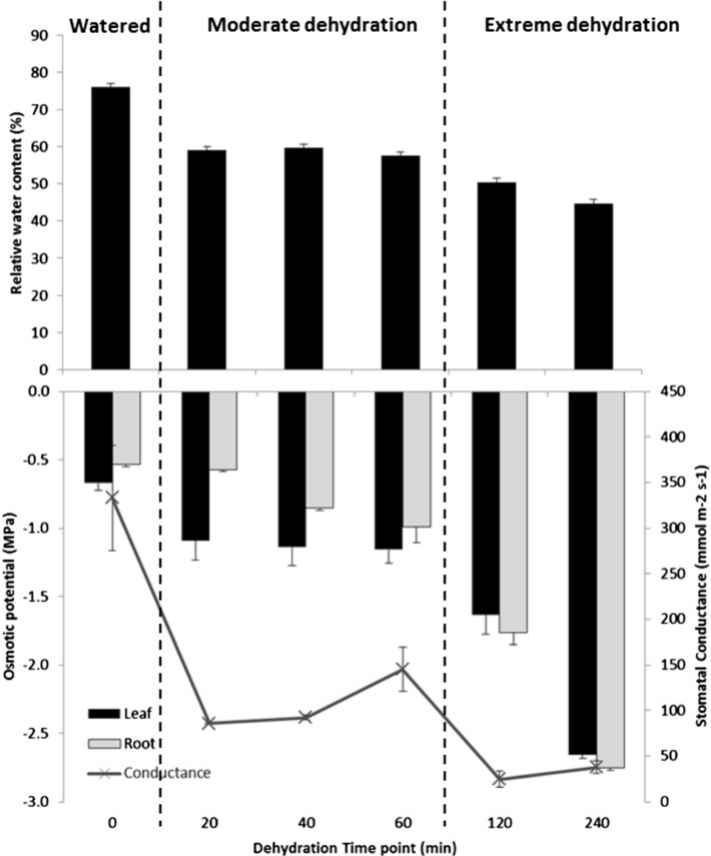
Relative water content (RWC, %), osmotic potential (MPa) and stomatal conductance (mmol/m/s) of leaves and osmotic potential of tobacco roots under moderate (20–60 min) and extreme (120–240 min) drought stress. Error bars (standard error) were calculated from three replicates.

Plants typically have to respond to multiple abiotic stresses simultaneously. This makes it difficult to characterize the signalling web that is associated with any one particular stress. To investigate drought responses we therefore performed experiments using hydroponic conditions where temperature, relative humidity, and the light regime were controlled. Tobacco cv ‘Burley 21’ plants were subjected to dehydration through air-drying for 20, 40, 60, 120 and 240 minutes by removing the plants from the hydroponics solution without touching the plants. This strategy ensured a strong and uniform response of the plants to drought stress, rapid harvesting, and a lack of wounding of the tissues. Plants showed symptoms of wilting after 20 minutes of dehydration. Further drought stress up to 240 minutes led to severe wilting. The tobacco plants were still alive after 240 minutes of dehydration because re-watered plants were able to fully recover showing expanded leaves. To determine the water status of the tobacco plants during the time course, osmotic potential and relative water content (RWC) were measured (Figure 1). The kinetics of RWC and osmotic potential changes differ with the greatest change in relative water content occurring in the first 20 minutes whereas the greatest change in osmotic potential occurs in the last 120 min. Stomatal conductance (SC) decreased rapidly (Figure 1) with a dramatic fall from 333 mmol/m/s (unstressed) to 86 mmol/m/s after 20 minutes indicating stomatal closure. Taken together, the physiological data show that stomatal closure is one of the most rapid responses of tobacco plants to drought stress and that the initial fall in osmotic potential occurs more rapidly in leaves than in roots.

### The metabolite level

**Figure 2:**
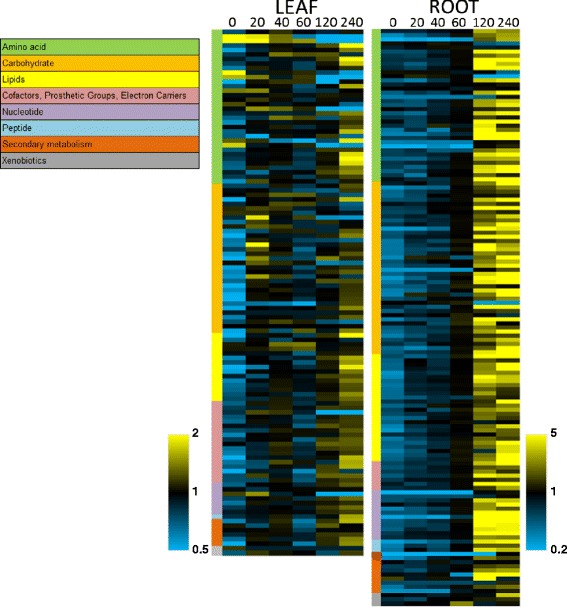
Heat map of relative changes in metabolites in leaf and root tissue during drought stress. Samples were analyzed by liquid chromatography/mass spectrometry (LC/MS, LC/MS2) and gas chromatography/mass spectrometry (GC/MS) platforms. The dataset comprised a total of 116 named biochemicals in leaves and 140 in root. Numbers indicate the time after the start of the experiment and the color scales the extent of changes in metabolites. Colored bars on the left of the heat map show the categories of metabolites.

A list of the notable changes in metabolite concentrations during drought is presented in Table 1 and they identify potential metabolic pathways that might be targeted for improving water stress responses. Levels of metabolites were determined by liquid chromatography/mass spectrometry (LC/MS, LC/MS2) and gas chromatography/mass spectrometry (GC/MS) platforms. The resultant dataset comprised 116 named biochemicals in leaves and 140 in root. Following log transformation and imputation with minimum observed values for each compound, Welch’s two-sample *t*-test was used to identify metabolites that differed significantly between experimental groups. Metabolites were identified that achieved statistical significance (p ≤ 0.05), as well as those approaching significance (0.05 < p < 0.1). An estimate of the false discovery rate (q-value) was also calculated (Additional file 1: Table S1). Our data show that drought stress caused considerable alterations in metabolite profiles in both leaf and root, even at the first time point (20 minutes) (Figure 2 and Additional file 1: Table S1) with changes in the biochemical profile of root tissues far more extensive than leaf (Figure 2). Nearly forty metabolites in the roots increased in level at least 10-fold during drought stress. By comparison, no metabolites in the leaves increased more than 5-fold. Principal Component Analysis (PCA) plots also reflect these trends (Additional file 2: Figure S1). In leaf the 60 min samples grouped more closely to the 0 min control than other groups, consistent with the biphasic nature of the response. In the root plot the time points in the first hour grouped relatively tightly, while the 120 and 240 min samples are well separated.

**Table 1:** **Features of the response of tobacco to drought stress mRNA, metabolite, and promoter levels**

	**Tissue**	**Observations**	**Comments**
UP-REGULATED GENES			
***NtERF187***	Leaves and roots	295-fold induced after 40 minutes in root. 23-fold in leaf after one hour.	Similar to Arabidopsis drought-inducible *AtERF53*, that regulates drought-responsive gene expression by binding to the GCC box and/or dehydration-responsive element (DRE) in the promoter of downstream genes. Overexpression of *AtERF53* driven by the CaMV35S promoter resulted in an unstable drought-tolerant phenotype.
***NtERF114*** **and** ***NtERF202***	Leaves	Rapid and transient up-regulation in leaves with a maximum of 37-55-fold induction after one hour.	Similar to Arabidopsis AtDREB1a/AtCBF3 which is involved in response to low temperature, drought, and abscisic acid.
***NtERF228***	Leaves and roots	Rapid and transient up-regulation in roots with a maximum of 127-fold induction at the first time point. Similar rapid and transient up-regulation in leaves.	Similar to Arabidopsis AtDREB1a/AtCBF3 which is involved in response to low temperature, drought, and abscisic acid.
***NtMYB149***	Roots only	Rapid up-regulation reaching 131-fold after 40 minutes. Not inducible in leaves.	Similar to AtMYB15. AtMYB15 is involved in ABA-, ethylene-, and JA-mediated signaling pathways, the response to salt stress, and the response to water deprivation
***NtERF218***	Leaves and roots	Rapid and transient up-regulation in both tissues.	Similar to Arabidopsis AtDREB1a/AtCBF3 which is involved in response to low temperature, drought, and abscisic acid.
***NtWRKY1***	Leaves and roots	Rapid and transient induction in leaves (28-fold). Low level induction in roots.	The apparent ortholog of AtWRKY33, which had been shown to play major roles in the response to stress including abiotic stress.
***NtERF75***	Leaves and roots	Strong (130-fold) late induction in leaves. Late and lower level induction in roots.	Similar to a member of the DREB subfamily A-6 in Arabidopsis. There are 8 members in this subfamily including RAP2.4.
***Ninja-family protein AFP3/ABI five-binding protein 3***	Leaves only	25-fold induced in leaves. Not induced in roots. (CHO_OF648xm02r1)	The Arabidopsis ortholog acts as a negative regulator of abscisic acid (ABA) responses and stress responses. Also called ABI five-binding protein 3.
***Glutathione peroxidases***		75-fold induced in leaves and 24-fold in roots. (CHO_OF6818xm12r1 and FG645026)	Control of H_2_O_2_ homeostasis, and linking ABA and H_2_O_2_ signaling in stomatal closure.
***Protein phosphatase 2C*** **genes**	Leaves and roots	Several genes up-regulated 20–70 fold in leaves and roots. (CHO_OF4760xf16r1 and EST EB442706)	Protein phosphatase PP2Cs acts as constitutive negative regulators of SnRK2 kinases whose autophosphorylation is required for kinase activity towards downstream targets in the ABA signaling network.
***NtUPLL1*** **and** ***NtUPLL2***	Leaves and roots	*NtUPLL1* is the most strongly up-regulated gene in leaves (291-fold) and both genes are strongly induced in both leaves and roots. (CHO_OF4952xo16r1 and CHO_OF569xh04r1)	Similar to the Arabidopsis U-Box E3 ubiquitin ligases AtPUB18 and AtPUB19 that negatively regulate ABA-mediated stomatal closure and drought stress responses.
***ABA 8'-hydroxylase CYP707A1***	Leaves and roots	Transiently up-regulated in the leaf (28-fold after 40 minutes). 6-fold in roots. (EST TC18468)	Play a major regulatory role in controlling the level of ABA in plants. Catabolizes ABA.
***5-Epiaristolochene 1,3-Dihydroxylase***	Leaves and roots	33-fold transiently induced in leaves. Not induced in roots. (EST AM821089)	Capsidiol is produced by Solanaceae plants in response to stresses such as pathogen or elicitor challenge.
***Cytochrome P450 CYP94C1***	Leaves and roots	Transiently up-regulated with a peak of 115-fold after 40 minutes in roots. Up-regulated later and less in leaves. (CHO_OF3036xp15r1, CHO_OF4654xf08r1 and CHO_OF3295xn18r1)	Arabidopsis cytochrome P450, CYP94C1 is involved in JA-Ile oxidation. The enzyme catalyzes catabolic turnover of JA-Ile. CYP94C1 and CYP94B3 catalyze successive oxidation steps in JA-Ile turnover.
***Cytochrome P450 CYP94B3***	Leaves and roots	Transiently up-regulated with a peak of 101-fold after 40 minutes in roots. Up-regulated later and less in leaves. (EST TC39596 and CHO_OF646xl21r1)	Arabidopsis cytochrome P450, CYP94C1 is involved in JA-Ile oxidation. The enzyme catalyzes catabolic turnover of JA-Ile. CYP94C1 and CYP94B3 catalyze successive oxidation steps in JA-Ile turnover.
***Anthocyanidin synthase***	Roots	61-fold induced after one hour of drought. (CHO_OF559xd02r1)	Catalyzes the penultimate step in the biosynthesis of anthocyanins
***UDP-glycosyltransferase 74B1***	Roots	54-fold induced after four hours of drought. (CHO_OF354xn10f1)	Involved in glucosinolate biosynthesis.
***Inositol polyphosphate 5-phosphatase***	Roots	35-fold after four hours of drought. (EST AM835516)	Predicted to modulate the phosphoinositide pathway, ABA levels and drought responses.
***NtWRKY69, NtWRKY3, NtWRKY10,*** **and** ***NtWRKY12***	Leaves (*NtWRKY3* and *69*) and roots (others)	All show early induction (20–40 minutes)	Apparent Solanaceae-specific induction of genes in Group IId. Tomato *SlWRKY10* is also induced by drought in leaves. Potential genes for improvement of Solanaceae drought responses.
DOWN-REGULATED GENES			
***Heat shock proteins HSF25*** **and** ***HSP40/DnaJ***	Roots	*HSF25-like* gene down-regulated 39-fold in roots only. *HSP40/DnaJ*-like gene 16-fold down-regulated in roots only. (CHO_OF623xn12f1 and EST AM780669)	Function in unfolded protein binding, heat shock protein binding.
***bZIP102***	Leaves	mRNA level goes down 11-fold in leaves	Closest Arabidopsis proteins are AtbZIP34 and AtbZIP61. Function unclear.
METABOLITES			
**4-hydroxy-2-oxoglutaric acid (KHG)**	Roots	Rapid early increase and 70-fold increase by 4 hours.	Possible novel mechanism to restart respiration upon water availability after drought. Appears specific to tobacco/Solanaceae as there is no increase in level during drought in soybean.
**Mannitol and trehalose**	Roots	Later time points were marked by a sharp increase in mannitol and trehalose.	Act as an osmoprotectants (compatible solute).
**Galactinol and Raffinose**	Leaves	In the leaf, galactinol and raffinose were undetectable until the final 240 min time point, suggesting an activation of the pathway due to the stress.	The raffinose pathway can provide osmolytes in situations of low water potential.
**Oxidized glutathione (GSSG) and dehydroascorbate**	Roots and leaves	GSSG levels increase 12-fold in roots. Dehydroascorbate levels double in leaves.	The glutathione-ascorbate cycle detoxifies hydrogen peroxide which is a reactive oxygen species and the cycle is activated in tobacco as a response to drought.
**γ-aminobutyrate (GABA)**	Roots	GABA levels increase 7.8-fold in roots.	The GABA shunt is a stress response pathway, the functions of which include controlling cytoplasmic pH, maintaining C/N balance by converting glutamate in the cytosol to succinate in the TCA cycle, and aiding in oxidative stress protection by generating NADH and succinate.
**Glycine and serine**	Leaves	Dramatic reduction of glycine and serine levels in leaves to 2-4% of initial values.	Tobacco tissues down-regulate photorespiration during drought as a mechanism to reduce the accumulation of toxic ammonia.
**Inosine**	Roots	Increases nearly 50-fold.	Probable nucleotide salvage pathway to recycle nucleosides. Inosine is formed by the deamination of adenosine.
HORMONES			
**ABA (abscisate/abscisic acid)**	Roots and leaves	The ABA concentration increased 8-fold after four hours in root tissue. ABA 8'-hydroxylase CYP707A1 genes are strongly and transiently up-regulated in the leaf. Many ABA responsive genes are up-regulated in both tissues. Components of ABA signaling such as protein phosphatase 2C genes are up-regulated.	ABA clearly plays a central role in regulating drought responses in tobacco.
**JA (Jasmonate)**	Roots	All of the biosynthetic enzyme genes in the JA biosynthetic pathway are rapidly and coordinately up-regulated in roots. At the metabolite level, there was a biphasic increase in N-delta-acetylornithine, which rises in response to JA. Many JA signaling components such as JAZ repressors are differentially regulated.	JA clearly plays an important role in the response to drought in tobacco, especially in the roots.
**Ethylene (Ethene)**	Roots and leaves	The biosynthetic enzyme genes in the ethylene biosynthetic pathway show up-regulation with strong tissue-specific up-regulation of ACC synthase genes and, to a lesser extent, ACC oxidase genes.	Ethylene plays a role in the regulation of drought responses.
PROMOTERS			
***NtWRKY69***	Leaves	Inducible by drought. Expression progresses upwards from the root and is initially in the vascular tissue before expression in all of the leaf. Also inducible by cold and possibly wounding.	Drought inducible promoter for leaf-inducible expression. ABA independent. Expression initially follows the vascular tissue upwards from the roots before spreading into all leaf cells. Contains three potential bHLH binding sites (CANNTG), one W box (TTGACT), one MYB binding site (CGGTCA).
			One of the Group IId genes that our data suggest may be part of a Solanaceae-specific response to drought.
***NtWRKY3***	Leaves	Inducible by drought. Also inducible by cold and possibly wounding	Drought and cold inducible promoter. One of the Group IId genes that our data suggest may be part of a Solanaceae-specific response to drought.
***NtWRKY70***	Leaves	Inducible by drought. Also inducible by cold and wounding	Drought, wound, and cold inducible promoter.
***NtUPLL2***	Leaves	Inducible by drought. Also inducible by cold and possibly wounding	Drought and cold inducible promoter.
***NtGolS***	Leaves	Inducible by drought. Also inducible by cold and wounding	Drought, wound, and cold inducible promoter.

The first column contains up- and down-regulated genes at the mRNA level, metabolites, hormones, and promoters that are prominent features of drought stress responses in tobacco. The second column describes which tissue(s) are involved. The third column details our observations in this report. The fourth column discusses the observations in a wider context. Transcription factor names are taken from the TOBFAC database.

It is striking that the levels of many metabolites increased relative to the time zero control (Additional file 1: Table S1). The few compounds which trend down over time did not appear random. They were mostly in the amino acid category and several were directly related to nitrogen metabolism (e.g. asparagine, aspartate) or were associated with the photorespiration pathway (glycine, serine), which can produce ammonia. Also, the one compound in nucleic acid metabolism which trended down was also a nitrogen storage molecule, allantoin. These patterns in the nitrogen pathways suggest impending nitrogen toxicity.

### Compatible solute accumulation

**Figure 3:**
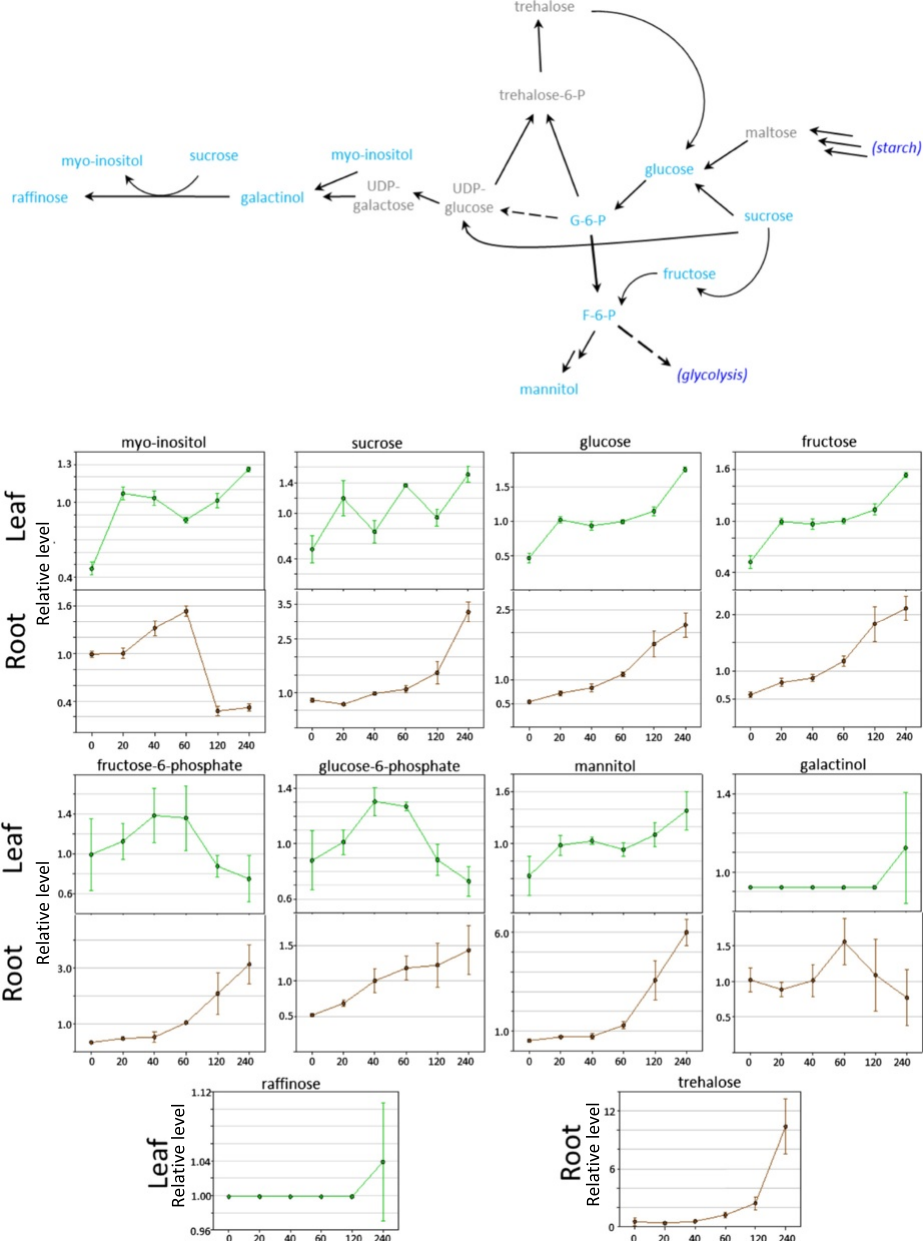
Changes in the sucrosyl-oligosaccharide/raffinose pathway during drought stress. The pathway is shown together with the relative levels of selected metabolites in the pathway. Error bars represent +/− one standard deviation. Green lines denote changes in leaves and brown lines changes in roots. X-axes numbers represent thedehydration time points in minutes. Y-axes values show the relative levels of the metabolite normalized to the median.

Our data showed evidence of drought and oxidative stress responses, as indicated by increased levels of compatible solutes such as sugar alcohols, amino acids, and oligosaccharides, as well as oxidative products of reactive oxygen species (ROS) remediation. The sucrosyl-oligosaccharide pathway, also known as the raffinose pathway, serves to transport and store carbon, and the resulting compounds can serve as osmolytes in situations of low water potential. In the leaf galactinol and raffinose were undetectable until the final 240 minutes time point, suggesting an activation of the pathway due to water stress (Figure 3). A decline in hexose-phosphates (glucose-6-P, fructose-6-P) and an increase in mannitol were also consistent with a net flow of carbon into the compatible solute forms (Figure 3). In roots there was a transient increase of galactinol and myo-inositol, but then a decline at later time points. However, the later time points were marked by a sharp increase in mannitol and trehalose, other compatible solutes often associated with drought stress. It may be that roots follow a different regulatory strategy, or the kinetics of carbon redistribution may be different in roots and leaves.

### Stress related compounds

Increases in several other stress related compounds were apparent, including oxidized glutathione (GSSG), threonate and dehydroascorbate (both products of ascorbate oxidation) (Additional file 1: Table S1). The glutathione-ascorbate cycle detoxifies hydrogen peroxide which is a reactive oxygen species. The cycle involves the antioxidant metabolites GSSG and dehydroascorbate and our data show that the glutathione-ascorbate cycle is being activated in tobacco as a response to drought stress.

### The GABA shunt

**Figure 4:**
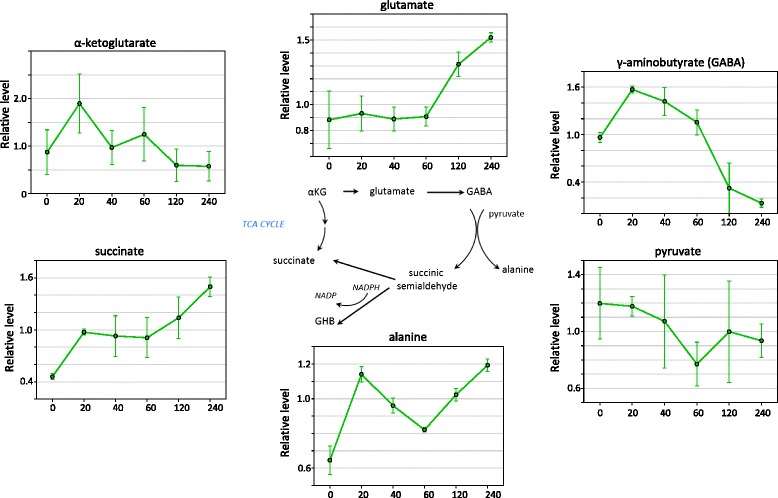
Changes in the GABA Shunt during drought stress in leaves. The pathway is shown together with the relative levels of selected metabolites in the pathway. Error bars represent +/− one standard deviation. X-axes numbers represent dehydration time points in minutes. Y-axes values show the relative levels of the metabolite normalized to the median.

The GABA shunt is a well-studied stress response pathway, the functions of which include controlling cytoplasmic pH, maintaining C/N balance by converting glutamate in the cytosol to succinate in the TCA cycle, and aiding in oxidative stress protection by generating NADH and succinate. Here we observed a rapid but transient increase in GABA in leaves, followed by a dramatic reduction after 120 min. In roots GABA slightly increased over the first hour, but then rose sharply after 120 minutes (Figure 4). This contrast in kinetics between tissues was similar to the patterns observed for other oxidative stress markers, such as glutathione, threonate, and dehydroascorbate.

### Nitrogen metabolism and maintenance of C/N balance

Nitrogen metabolism and maintenance of C/N balance is a key aspect not only of normal growth and development but also stress management in plants. A strong demand for carbon diversion into oligosaccharides (e.g. raffinose pathway), disaccharides (e.g. trehalose), or sugar alcohols (e.g. mannitol, sorbitol) as osmolytes can lead to excess nitrogen in the system (as toxic ammonia). Photorespiration also generates ammonia, resulting from the conversion of glycine to serine, and of serine to beta-hydroxypyruvate. The normal response to excess ammonia is to capture it through the nitrogen assimilation pathways (glutamine synthase, glutamate dehydrogenase, glutamine oxoglutarate aminotransferase) and sequester and/or transport excess nitrogen as asparagine and other nitrogen rich compounds such as allantoin. Failure of this mechanism can lead to ammonia toxicity and cell damage [44]. In our study, an increase in glutamate and glutamine at the late time points in both leaves and roots, suggested increased assimilation. However, at the same time there was a dramatic drop in aspartate levels, the precursor of asparagine, and a decrease in asparagine and allantoin (Additional file 1: Table S1). This suggests that the conversion of oxaloacetate to aspartate by aspartate aminotransferase (with the amino group donated by glutamate) may be limiting the flow of excess nitrogen into asparagine, and may contribute to ammonia toxicity.

### The photorespiration pathway

**Figure 5:**
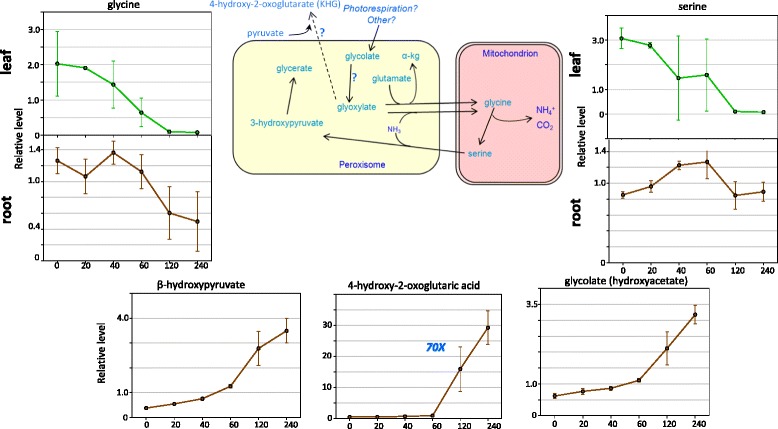
Changes in the Photorespiration pathway during drought stress. The pathway is shown together with the relative levels of selected metabolites in the pathway. Error bars represent +/− one standard deviation. Reactions are shown in the mitochondria, the peroxisomes, and the cytoplasm. Green indicates results for leaf and brown for roots. X-axes numbers represent the dehydration time points in minutes.Y-axes values show the relative levels of the metabolite normalized to the median.

The photorespiration pathway can generate ammonia, and the two amino acids involved in the pathway (glycine and serine) showed a common pattern contrary to the general amino acid accumulation (Figure 5). They both decreased during drought stress in leaves and roots, most dramatically in leaves. This suggests that tobacco tissues down-regulate photorespiration during drought stress as a mechanism to reduce the accumulation of toxic ammonia. This phenomenon is well documented [4]. The behaviour of another metabolite, 4-hydroxy-2-oxoglutaric acid (KHG, also known as 2-hydroxy-4 oxopentanedioic acid, 4-Hydroxy-2-ketoglutarate, 2-Keto-4-hydroxyglutarate and other names), may be related to this shut down of photorespiration. The levels of KHG show the greatest increase, relative to controls, of any compound in the study (Figure 5 and Additional file 1: Table S1). KHG was undetectable in leaves but there was a rapid 20-fold increase in KHG levels in root tissues between 60 and 120 minutes of drought stress. After four hours, KHG levels reached a striking 70-fold higher than control root tissue. It is not clear what the role of KHG is and further plant-based work is required. KHG can be broken down to pyruvate and glyoxylate by the corresponding aldolase and in other systems the aldolase plays a role in respiratory metabolic pathways, for example during respiration resumption during the termination of the *E.coli* SOS response [5]. It is possible that KHG accumulates in tobacco during drought stress and is then broken down into pyruvate and glyoxylate when water is available again. This would represent a novel mechanism that is used by tobacco plants to restart respiration upon water availability after drought.

### The mRNA level

Gene expression at the mRNA level was monitored using a Nimblegen 12 x plex custom oligo array. The oligo array contained multiple 60mer probes to individual gene sequences from three different sources. Firstly, 40,000 individual genomic survey sequence reads (GSSs) from the Tobacco Genome Initiative project with the highest E-value hits to proteins in the database, secondly, all TOBFAC transcription factors [52], and thirdly the Version 3.0 DFCI Tobacco Gene Index EST sequences. This ensured a wide coverage of tobacco genes (Additional file 3: Table S2 and Additional file 4: Table S3). We previously produced a tobacco MapMan mapping that can be used directly with our NimbleGen oligoarray to visualize the oligo array data [39]. Using these data, we were able to identify genes that showed significant increases or decreases at the mRNA level during drought stress and correlate these data with changes at the metabolite level.

**Figure 6:**
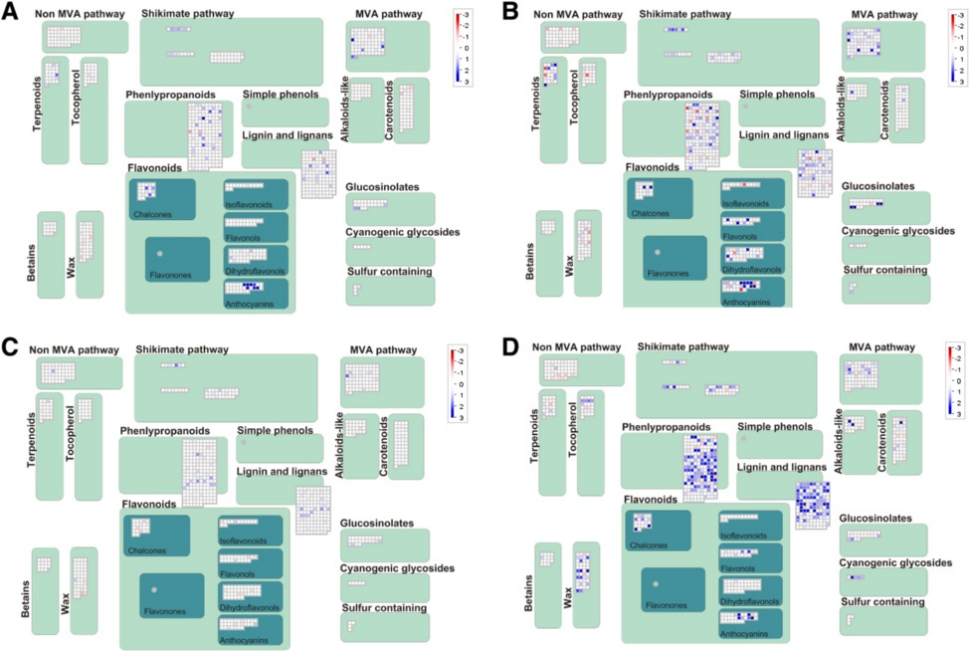
MapMan visualization of genes involved in secondary metabolism. Pathway visualization was performed using our tobacco MapMan mapping [39]. Blue denotes genes that are up-regulated and red genes that are down-regulated. The color scheme is shown in a rectangle using a log 2 scale. The most intense color therefore represents changes of 8-fold or more. **A**. Changes in root after 40 minutes. **B**. Changes in root after 4 hours. **C**. Changes in leaf after 40 minutes. **D**. Changes in leaf after 4 hours.

A MapMan visualization of genes involved in secondary metabolism during drought shows that there are major differences between leaf and root tissues at the mRNA level (Figure 6). In roots, genes involved in anthocyanin production were among the most strongly up-regulated genes. Also up-regulated are genes involved in the biosynthesis/catabolism of glucosinolates, chalcones, flavonols, and terpenoids (Figure 6). In leaves, genes involved in anthocyanin, chalcone, phenylpropanoid, lignin and wax metabolism are strongly up-regulated.

In leaf, after forty minutes of dehydration, approximately two hundred genes showed at least 8-fold induction in mRNA levels (Additional file 5: Table S4). This number increased to over eight hundred after four hours. The up-regulated genes at the early time points included a large percentage of signalling molecules such as transcription factors, protein kinases, F Box proteins, and phosphatases (Additional file 5: Table S4). Over the time course of drought stress, the most strongly up-regulated gene in tobacco leaves also encodes a signalling molecule, a U-Box E3 ubiquitin ligase that we have called Ubiquitin Protein Ligase Like 1 (NtUPLL1) (Additional file 5: Table S4). The level of *NtUPLL1* mRNA increases 52-fold after 40 minutes and over 290-fold by the four hour time point. NtUPLL1 and the closely related NtUPLL2 are similar to the Arabidopsis U-Box E3 ubiquitin ligases AtPUB18 and AtPUB19 that regulate ABA-mediated stomatal closure and drought stress responses [58].

At the later time points in leaf, the list of up-regulated genes includes many biosynthetic enzyme genes that produce osmoprotectants such as raffinose synthase and galactinol synthase and this reflects the observed changes in metabolites (Figure 3). Other well documented drought stress-inducible genes such as dehydrins, aquaporins, and LEA proteins also show induction. This includes glutathione peroxidase genes (Additional file 5: Table S4) which are considered the main enzymatic defence against the oxidative destruction of membranes [3,67]. Glutathione peroxidases may play dual roles, the first in the control of H_2_O_2_ homeostasis, and the second in linking ABA and H_2_O_2_ signalling in stomatal closure, thereby regulating transpiration [43]. This observation is consistent with an increased flow through the glutathione-ascorbate cycle at the metabolite level. We observed a 33-fold increase in 5-Epiaristolochene 1,3-Dihydroxylase mRNA levels. 5-Epiaristolochene 1,3-Dihydroxylase catalyses the formation of capsidiol, a sesquiterpene produced by Solanaceous plants in response to stresses [68]. Several glutamate decarboxylase (GAD) genes were up-regulated at the mRNA level and this represents a correlation between increases in the metabolite and mRNA levels. GAD catalyses the decarboxylation of glutamate to GABA and CO2 and these data show that the GABA shunt stress response pathway is up-regulated by drought stress in tobacco at the mRNA level and this is subsequently reflected in an increase in GABA at the metabolite level.

In roots at the earliest time point, the up-regulated genes were again predominately signalling molecules including AP2/ERF and MYB transcription factors, MAP kinases and calcium dependent protein kinases (Additional file 5: Table S4). Calmodulin-related genes are also prominent in the list of up-regulated genes. Other strongly induced genes include two *CYP94C1* and *CYP94B3* genes that catalyse two successive oxidation steps in JA-Ile catabolic turnover. Both JA and ethylene biosynthetic enzyme genes were strongly induced, as were several protein phosphatase 2C genes. Similar to leaf tissues, at later time points, the list of up-regulated genes includes many biosynthetic enzymes genes that produce osmoprotectants. Other notable genes include an *anthocyanidin synthase* gene (61-fold induced after one hour) that catalyses the penultimate step in the biosynthesis of anthocyanins [6] and a *UDP-glycosyltransferase 74B1*-like gene (54-fold induced after four hours) involved in glucosinolate biosynthesis. These genes are potential targets for engineering secondary metabolism (anthocyanins and glucosinolates, respectively) and drought tolerance (Table 1).

The phosphoinositide (PI) pathway is an important regulator of cellular functions [12] and the PI pathway and inositol-1,4,5-trisphosphate are implicated in plant responses to stress [45]. An *inositol polyphosphate 5-phosphatase*-like gene (AM835516) is strongly up-regulated in roots. Arabidopsis plants overexpressing a mammalian *inositol polyphosphate 5-phosphatase* lost less water and exhibited increased drought tolerance. The onset of drought stress was delayed in these transgenic plants and ABA levels increased less than in wild-type plants [45]. The tobacco inositol-1,4,5-trisphosphate 5-phosphatase enzyme is therefore predicted to modulate the phosphoinositide pathway, ABA levels and drought responses and could be an excellent tool to improve drought stress responses in the Solanaceae (Table 1).

### Plant hormones

Both the metabolomics and transcriptomics data show roles for the plant hormones ABA, JA, and ethylene in the response to drought stress in tobacco (Additional file 6: Figure S2). At the metabolite level, ABA (abscisate) increased 8-fold after four hours in root tissue. This observed kinetics of ABA increase suggests that many immediate early genes are activated before any increase in ABA levels (Additional file 5: Table S4 and Additional file 7: Table S5) and this is consistent with observations that early responses may by triggered by a rapid hydraulic signal in an ABA-independent manner [8,9]. There was also a biphasic increase in N-delta-acetylornithine, which has been shown to rise in response to methyl-jasmonate. At the transcriptome level, MapMan visualization revealed that ABA, JA, and ethylene all play major roles in regulating drought stress (Additional file 6: Figure S2).

The biosynthetic enzyme genes in the pathway that produces JA were all up-regulated in tobacco root tissue and to a lesser extent in leaves (Additional file 6: Figure S2). The biosynthetic enzyme genes in the ethylene biosynthetic pathway are also up-regulated (data not shown). We found numerous other differentially regulated genes that provide insights into ABA and JA signalling in drought stressed tobacco plants. A ninja-family protein *AFP3/ABI five-binding protein 3* gene is strongly induced in leaves at later time points. NINJA proteins acts as negative regulators of ABA responses and stress responses [19] and are part of a repressor complex that negatively regulates JA signalling [1]. Interestingly, two closely related cytochrome P450 CYP94 genes (*CYP94C1* and *CYP94B3*) are strongly and transiently induced in root tissue (Table 1). In Arabidopsis, these two enzymes catalyse two successive oxidation steps in JA-Ile catabolic turnover [23] and these data suggest that drought stress in tobacco activates the genes for a major catabolic route for turning over JA-Ile. Our data show that not only genes for JA catabolism appear to be up-regulated but also genes for ABA catabolism. ABA 8'-hydroxylase (*CYP707A1*) genes are strongly and transiently up-regulated in the leaf (Table 1) and encode key enzymes in controlling the level of ABA in plants [36] with the hormonal action of ABA being controlled by the precise balance between its biosynthesis and catabolism.

### Transcription factors

Transcriptional reprogramming is a central component of the response to drought stress. For this reason we investigated the transcription factors (TFs) that are up-regulated by drought stress. A MapMan visualization of the TF genes present on the oligo array is presented in Additional file 8: Figure S3. It highlights the tissue-specific differences in the transcriptional regulation of TFs during drought stress in tobacco.

We produced a non-redundant list of the thirty most highly induced TF genes in leaf and root tissues at an early (40 minutes) and late (four hours) time point (Additional file 7: Table S5). All TF genes are named according to the TOBFAC database nomenclature [52]. After 40 minutes of drought stress in root tissues, AP2/ERF TFs were the main group of up-regulated genes (18 genes), followed by MYB, bHLH, WRKY, C2H2 zinc finger, JAZ/TIFY, and bZIP (Additional file 7: Table S5). The most highly induced TF gene was *NtERF187* (295-fold) which encodes a protein similar to Arabidopsis AtERF53 (Table 1) which plays a role in drought-regulated gene expression [7,24]. The second most highly induced gene was *NtMYB149* which is similar to Arabidopsis *AtMYB15. AtMYB15* is involved in ABA-, ethylene-, and JA-mediated signalling pathways, the response to salt stress, and the response to water deprivation. Overexpression of *AtMYB15* confers enhanced sensitivity to ABA and improved drought tolerance in Arabidopsis [14] and this suggests that *NtMYB149* is a good candidate for improving drought tolerance in the Solanaceae. The next highest induced TF gene was *NtERF13* which is in the same clade as CBF1, CBF2, and CBF3 that play roles in cold and dehydration stress. Clearly some of the major TF nodes in drought stress signalling are conserved between tobacco and Arabidopsis and probably across dicot plants as a whole. After four hours, the same three TF genes (*NtERF187, NtERF13,* and *NtMYB149*) were still the most highly induced in roots. However, in addition three NAC TFs and three WRKY TFs are also among the most highly induced genes at this time point (Additional file 7: Table S5).

In leaf, there were major differences in the TF mRNA profile compared to root (Additional file 7: Table S5). Although more AP2/ERF genes were up-regulated than other TF families (nineteen genes), WRKY genes (seven) constituted a considerable proportion of the list of thirty most strongly up-regulated genes. The two most strongly up-regulated genes after 40 minutes were the closely related *NtERF202* and *NtERF114* (also called *ACRE111A* and *ACRE111B*) (Table 1). Both are similar to Arabidopsis *CBF3/DREB1A. NtWRKY1* is among the most highly induced genes during the early response (Table 1). Prominent among the up-regulated WRKY TF genes, *NtWRKY1* is the apparent orthologue of *AtWRKY33*, which has been shown to play major roles in the response to stresses including abiotic stress [28]. The late time point in leaves revealed a much more diverse profile of differentially regulated TF genes with representatives from the AP2/ERF, WRKY, JAZ/TIFY, NAC, bZIP, MYB, GRAS, homeodomain, and AUX-IAA families.

**Figure 7:**
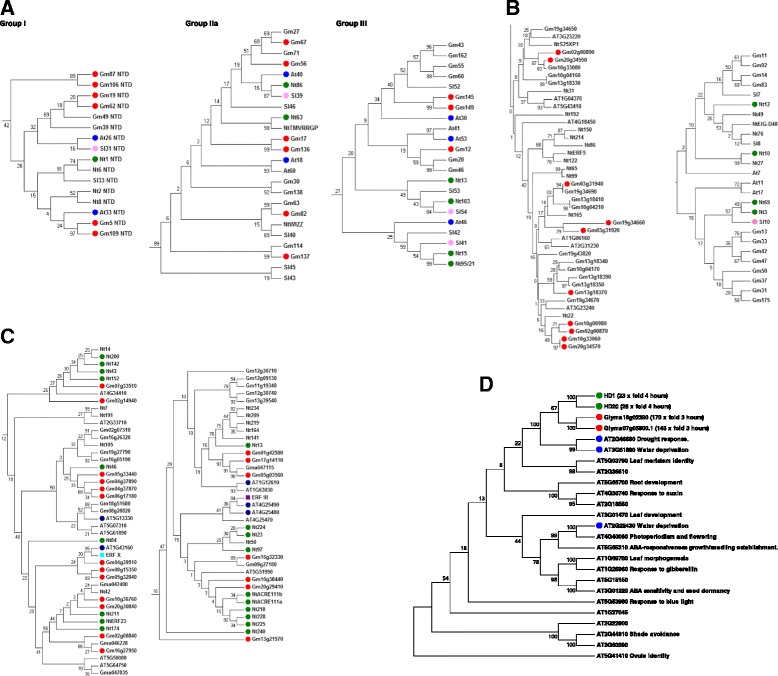
Drought-inducible transcription factor genes in the WRKY, HDZIP, and AP2/ERF families. **A**. Core responses in the WRKY transcription factor family **B**. Family/species-specific responses in the AP2/ERF and WRKY families **C**. Core responses in the AP2/ERF family **D**. Core responses in the HDZIP family. Drought stress inducibility or association with the GO term “Response to water deprivation” (GO:0009414) is denoted by red circles (soybean), blue circles (Arabidopsis), green circles (tobacco), and pink circles (tomato). Marker domains for Group III and X ERF subfamilies are shown as purple and light blue squares respectively. The evolutionary histories were inferred using the Neighbor-Joining method. The optimal trees are shown. The percentage of replicate trees in which the associated taxa clustered together in the bootstrap test (1000 replicates) is shown next to the branches. The evolutionary distances were computed using the Poisson correction method and are in the units of the number of amino acid substitutions per site. For the HDZIP transcription factors, induction by drought stress and the processes that the transcription factors are involved in are shown. AP2/ERF and homeodomain genes are shown by gene model name except for tobacco where the TOBFAC nomenclature is used. WRKY genes from Arabidopsis are shown by their commonly used names, soybean from our own analysis (Additional file 9: Table S6), tobacco genes were taken from TOBFAC and tomato genes from published sources.

One of our aims was to identify genes that might be used to improve drought stress responses, not just in tobacco but also in other members of the Solanaceae. We therefore compared drought stress inducible WRKY genes from soybean (Additional file 9: Table S6), Arabidopsis, tobacco, and tomato to determine commonalities that may represent core changes in transcription factor gene expression and differences that could be responsible for family/species-specific responses to drought stress. The drought stress analyses were obtained as follows: tobacco (this study, GEO accession GSE67434), soybean (GEO accession GSE49537), Arabidopsis [33,72] and tomato [20,25,38]. There are three major hotspots of drought stress inducibility in the WRKY phylogenetic tree. The first is in Group III with two sets of tobacco/tomato orthologues (*NtWRKY15/NtWRKY95/21/SlWRKY41* and *NtWRKY103/SlWRKY54*) and inducible Arabidopsis and soybean genes. The second is in Group I in a clade that includes *NtWRKY1* and *SlWRKY31.* In the data sets studied, *NtWRKY1* is the most strongly induced WRKY gene after 20 minutes of drought stress in tobacco leaves and *SlWRKY31* is the most strongly induced WRKY gene in drought stressed tomato leaves. This suggests that induction of *NtWRKY1/SlWRKY31*-like genes is a feature of drought responses in the Solanaceae and that these genes are good candidates for manipulating drought responses. The final hotspot is in Group IIa, where *NtWRKY86* and *SlWRKY39* are induced by drought stress as well as several Arabidopsis and soybean genes (Figure 7). Group IIa is a well-studied group and includes *AtWRKY18, AtWRKY40,* and *AtWRKY60* that play central roles in the responses to multiple stresses [54,65].

Interestingly, as well as similarities in TF gene induction our data suggest that there may be Solanaceae-specific drought stress inducibility of several Group IId WRKY genes from tobacco and tomato (Figure 7). Tobacco *NtWRKY3, NtWRKY10, NtWRKY12,* and *NtWRKY69*, together with *SlWRKY10* all show drought stress inducibility whereas under similar experimental conditions, no soybean IId genes showed this induced expression. In addition, the Arabidopsis IId genes *AtWRKY7*, *AtWRKY11*, and *AtWRKY17* have been shown to modulate transcription in response to pathogen challenge [29] but have also not been shown to play any role in responses to drought.

There are notable similarities in the ERF/AP2 family when we compared drought stress-inducible genes from tobacco and soybean with Arabidopsis genes that are annotated with the GO term “Response to water deprivation” (GO:0009414). As expected, a large number of genes from the DREB subfamily (Groups I, II, IV, and particularly III) are induced by drought stress in tobacco (Additional file 8: Figure S3). Group III (including the Arabidopsis genes AT4G25490/CBF1/DREB1B and AT4G25480/CBF3/DREB1A), is a hotspot with at least 20 out of 36 genes being associated with the response to drought stress (Figure 7). In the ERF subfamily, a small number of genes from Group VIII, a single Group VI gene and no Group VII genes showed inducibility by drought stress. By contrast, many Group IX and X genes showed induction. The functions of many ERF subfamily transcription factors in abiotic stress responses are largely unknown [42] and it was therefore of interest that Group X is a hotspot of drought stress responsive ERF/AP2 genes (Figure 7). Our data suggest that Group X ERF transcription factors play roles in the response to drought stress but unlike the well-studied DREB transcription factors, the exact roles of many remain to be determined.

We again observed apparent family-specific drought stress-inducibility of ERF/AP2 genes. In Subgroup 3 of the Group IX ERF genes there are no fewer than ten soybean ERF genes that are inducible by drought stress. Strikingly, not a single tobacco gene showed induction (Figure 7). This appears to be a family/species-specific role for soybean ERF transcription factors in the response to drought stress. This is particularly noticeable in the area of the phylogenetic tree that includes GLYMA10g00980, GLYMA02g00870, GLYMA10g33060, and GLYMA20g34570. *GLYMA20g34570* is induced 35-fold by drought stress in leaves and *GLYMA10g00980* is induced 128-fold making it one of the most highly up-regulated genes by drought stress in soybean leaves. By contrast, the most similar tobacco gene *NtERF22* (Additional file 10: Figure S4) shows no induction by drought stress whatsoever (Additional file 11: Table S7). This poses the question what the tobacco Subgroup 3 transcription factors may be doing and our previous work sheds light on this [57]. *NtERF5*, *NtERF165*, *NtERF22* and *NtS25XP1* have all been implicated in regulating jasmonate-inducible nicotine biosynthesis in tobacco [57]. Interestingly, nicotine biosynthesis occurs only in the Solanaceae, predominantly in tobacco, and in lower quantities in tomato, potato, eggplant, and green pepper. This suggests that family-specific differences in secondary metabolism are reflected in family-specific roles of some transcription factors.

There are also similarities in drought stress responsiveness in the homeodomain family. In tobacco, two HDZIP genes (*HD1* and *HD20*) are strongly up-regulated by drought stress in leaf and Figure 7 shows that the two orthologues in soybean (*Glyma16g02390* and *Glyma07g05800*) are also very strongly induced by drought stress. Strikingly, the two Arabidopsis orthologues of these genes (*AT2G46680/ HOMEOBOX 7* and *AT3G61890/HOMEOBOX 12*) have been shown to play roles in the responses to drought stress and our data suggest that orthologues of these HDZIP transcription factors regulate drought stress responses across dicot plants and possibly beyond.

### Promoters

To validate and characterize drought stress inducibility, several genes were selected for promoter:reporter analyses in transgenic tobacco plants. We chose five drought stress inducible genes, galactinol synthase (*NtGolS*), *NtUPLL2*, *NtWRKY69*, *NtWRKY70*, and *NtWRKY3*. An additional two promoters (*NtWRKY95*/21 and a *raffinose synthase*) have previously been validated in transgenic tobacco BY-2 cells [47] where they are inducible both by polyethylene glycol and jasmonate. Our choice of promoters was partly dictated by available promoter sequence. For example, promoter sequences of *NtWRKY86*, and *NtUPLL1* were not available.

**Figure 8:**
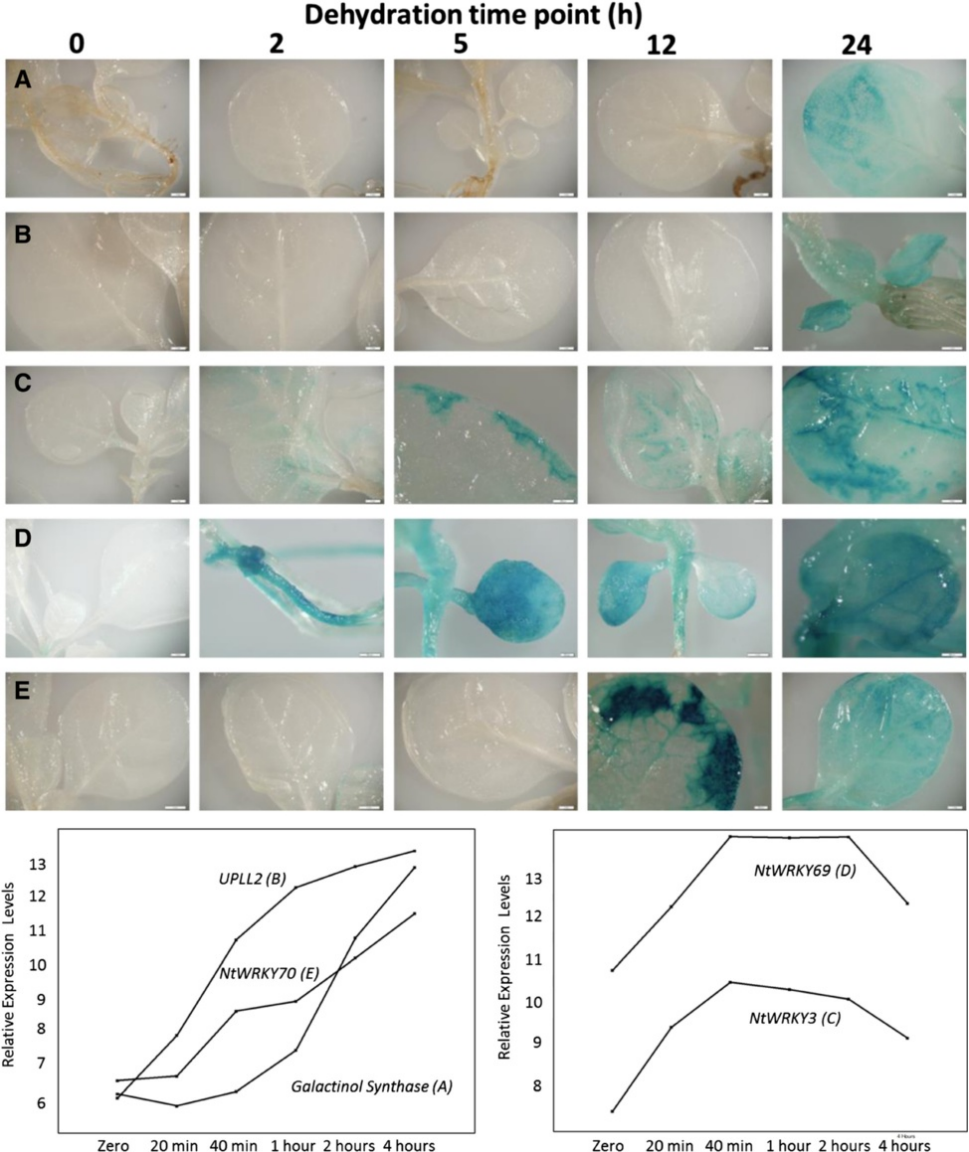
Activity of promoter-GUS reporter gene constructs in 4-week old transgenic tobacco plants at different drought stress time points. Inset line graphs show the expression profiles of the genes at varying dehydration time points at the mRNA level from oligo array analysis.

Transgenic tobacco plants containing promoter-GUS constructs were tested for reporter gene activity under dehydration, cold and wounding. Figure 8 shows GUS expression from the different promoters during a time course of drought stress. The *NtWRKY69* and *NtWRKY3* promoters directed early induction by drought stress in leaves with GUS activity observed as early as two hours after the start of dehydration (Figure 8). The kinetics of GUS activity directed by the promoters *in planta* was similar to the kinetics of mRNA accumulation observed using the oligo array. Both *NtWRKY3* and *NtWRKY69* show early increases in mRNA levels and these increases are reflected in early promoter activities *in planta*. In contrast, promoters from *NtWRKY70*, *NtGolS* and *NtUPLL2* directed later induction by drought stress. Again this correlates with mRNA levels that show major increases during the later stages of dehydration.

To determine whether these promoters also respond to other types of abiotic stress, the transgenic plants were subjected to cold and wounding (Figure 8). All promoter:reporter transgenic lines showed inducibility by cold treatment (Figure 8). In addition the promoters also showed varying degrees of inducibility by wounding (Figure 8). *NtWRKY3*, *NtWRKY69*, *NtWRKY70*, *NtGolS* and *NtUPLL2* are all therefore responsive to multiple abiotic stresses and are components of interconnected abiotic stress-responsive signalling webs.

**Figure 9:**
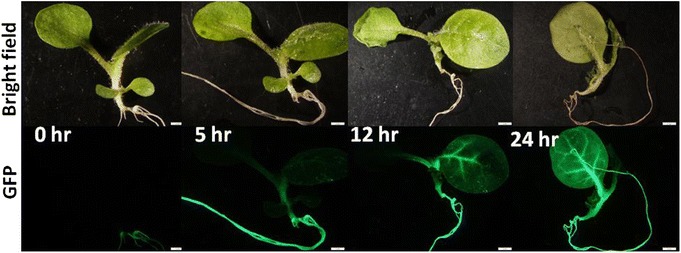
*NtWRKY69* promoter activity in 4-week old transgenic tobacco plants during drought stress. ProNtWRKY69:GFP-containing plants illustrate the progression of promoter activation in stems and then leaves during drought stress.

We chose to further study the temporal and spatial activation of the *NtWRKY69* promoter in more detail because it is one of the Group IId genes that our data suggest may be part of a Solanaceae-specific response to drought stress (Figure 7). Using GFP as the reporter gene enabled monitoring of promoter activity over the course of drought stress and a remarkable activation of the *NtWRKY69* promoter was observed (Figure 9). In the absence of drought stress, little or no activity was seen in the leaves. After the start of drought stress, promoter activity started at the base of the stem and then moved up the stem and into the leaf. Promoter activity was subsequently found in the vascular tissue of the leaf and then eventually spread out into the non-vascular tissue. This suggests that a signal from the roots is moving through the stem and then into the leaves through the vascular tissue. Whether this signal is hormonal (such as ABA and/or JA) or hydraulic is unclear. To determine whether the signal might be ABA, 20 μM ABA was sprayed onto the leaves of the transgenic tobacco. The treated leaf areas showed no GFP activity. As a control, the ABA-treated plants were then subjected to dehydration and inducible promoter activity was again seen (data not shown). This suggests that the promoter of *NtWRKY69* is not responsive to exogenous ABA and therefore that NtWRKY69 functions in an ABA-independent pathway. Taken together, our data suggest roles for JA-dependent, ethylene-dependent, ABA-dependent and ABA-independent pathways in the water stress induced signalling web in tobacco.

## Discussion

### Core and family/species-specific features of the drought stress response in tobacco

The responses of many plant species to drought stress have been extensively studied [13,30,33,59,61,66,71]. Several hormones play major roles in drought responses, with ABA being a major player. ABA plays a major role in drought signalling and water stress causes an immediate hydraulic signal that triggers ABA biosynthesis [49]. ABA perception occurs by a mechanism where binding of ABA to the ABA receptors RCARs/PYR1/PYLs results in inactivation of type 2C protein phosphatases including ABI1 and ABI2. These type 2C protein phosphatases appear to function as co-receptors and they inactivate SNF1-type kinases. This results in activation of ABA-dependent gene expression [49]. To understand the mechanisms involved in the response to drought stress, it is necessary to understand the changes in the plant cell at many interconnected levels. We therefore sought to correlate changes at different levels by using the same plant samples for physiological, mRNA, and metabolite analyses. We have also been able to suggest common features of plant drought stress responses and additonally features that may be unique to tobacco or members of the Solanaceae. Comparative analyses of large omics data sets are hampered by the varying experimental setup [32]. However, the data set from soybean (GEO accession GSE49537) was produced under very similar conditions of hydroponics/dehydration to the tobacco data set and facilitates direct comparisons between a member of the Solanaceae and a legume. Our comparisons, together with recent reports from other groups [13,30,59,61,66,71] lead us to suggest that the following represent some of the core metabolic changes of plants to drought stress.

1. The production of glutathione and tocopherol as anti-oxidants.
2. Ammonia detoxification.
3. Increases in amino acids that act as osmolytes.
4. Activation of the raffinose pathway to produce osmolytes.
5. Regulation of nitrogen metabolism (asparagine, allantoin and glutamine).
6. Activation of the GABA Shunt to control cytoplasmic pH, maintain C/N balance and protect from oxidative stress.

The suggestion concerning the detoxification of ammonia is based mainly on the present study and the study by Oliver *et al*. [44] on desiccation tolerance in *Sporobolus stapfianus*. These studies point to ammonia detoxification as a core metabolic process during water stress. The ammonia itself would appear to come from a strong demand for carbon diversion into oligosaccharides (e.g. raffinose pathway), disaccharides (e.g. trehalose), or sugar alcohols (e.g. mannitol, sorbitol) and these osmolytes can lead to excess nitrogen in the system (as toxic ammonia). Photorespiration also generates ammonia, resulting from the conversion of glycine to serine, and of serine to beta-hydroxypyruvate. The exact role of ammonia detoxification in water stress responses and the mechanisms involved require further study.

In addition to these core responses, there are other responses that appear species/family specific. For example KHG, the most strongly induced metabolite in tobacco is not induced by drought stress in all plants. Our data show that although KHG is detected in drought stressed soybean roots, it is not induced by drought stress (Table 2). This shows that major responses to drought stress are family specific and not part of the core responses to drought stress.

**Table 2:** **Relative increases in 4-hydroxy-2-oxoglutaric acid levels in tobacco and soybean during dehydration**

**Tobacco root**					
	20 min/0 min	40 min/0 min	60 min/0 min	120 min/0 min	240 min/0 min
4-hydroxy-2-oxoglutaric acid	1	1.5	1.98	38.08	70.12
**Soybean root**					
	30 min/0 min	60 min/0 min	120 min/0 min	180 min/0 min	300 min/0 min
4-hydroxy-2-oxoglutaric acid	1.56	0.94	1.55	2.05	1.78

A global unbiased metabolic profiling platform from both plant species was used as previously described (Evans et al. 2009 [16]). 4-hydroxy-2-oxoglutaric acid was identified by comparison to library entries of purified standards. The values show the fold increases relative to control unstressed plants.

Similar to the metabolite level, at the mRNA level transcription factor genes show both core changes and family specific responses in transcription factor gene expression. Core changes include up-regulation of members of the Group I, IIa, and III WRKY, Group III and X AP2/ERF, and HDZIP (Figure 7) transcription factors. Family-specific changes include Group IX AP2/ERF genes from soybean that are up-regulated by drought stress and Group IId WRKY genes from tobacco and tomato that likewise show drought stress inducibility. Taken together, it is likely that the family-specific changes in metabolism are regulated, at least in part, by family-specific changes in transcription factor activity. A challenge for the future is to further establish the roles of individual transcription factors in regulating these family-specific changes in metabolism. However, our data has already provided insights into this. Many Group IX Subgroup 3 ERF transcription factors in soybean appear to play roles in the response to drought stress (Figure 7), whereas their counterparts in tobacco appear not to regulate drought stress responses but rather the Solanaceae-specific increase in nicotine biosynthesis as a response to herbivory [57]. These observations provide a framework for the parallel study of family-specific metabolic changes and family-specific differences in transcription factor function. The large nature of our data set has precluded a study of all transcription factor families, but we are confident that similar core and family-specific changes are to be found outside of the AP2/ERF, WRKY, and homeodomain families. Indeed, an elegant recent study has suggested that there are core environmental stress response genes that are coordinately regulated not only as a result of drought stress but also more widely as a response to abiotic stresses [22].

The very high accumulation of KHG in tobacco roots strongly suggests that it is playing a role in combating drought stress but it is not currently clear what this mechanism is because KHG is poorly studied in plants. KHG can be broken down to pyruvate and glyoxylate by the corresponding aldolase (Figure 5) and in other systems the aldolase plays a role in respiratory metabolic pathways. One hypothesis comes from studies of the *E. coli* SOS response where KHG accumulates to promote respiration resumption during the termination of the *E. coli* SOS response [5]. We suggest a similar mechanism where KHG accumulates in tobacco during drought stress and is then broken down into pyruvate and glyoxylate when water is available again. This would represent a novel mechanism that is used by tobacco plants to restart respiration upon water availability after drought. Alternatively, the rise in activity of glutamine oxoglutarate aminotransferase (GOGAT) and it is possible that KHG serves as a scavenger for ferredoxin, NADP or the detoxification of NO_2_, which could have accumulated due to the dehydration. This and/or other functions of KHG in plants require further study.

### Targets and new strategies for Solanaceae crop improvement

Table 1 lists some of the noteworthy features of the tobacco response to drought stress at the gene, metabolite, plant hormone, transcription factor, and promoter levels. Many of these represent potential targets for the improvement of Solanaceae crop plants such as tomato and potato. These systems biology data will prove valuable when designing strategies for crop improvement. For example, a drought stress-inducible promoter can be used to drive a transgene that affects the accumulation of identified key metabolites. An inducible promoter will restrict transgene expression to when and where it is needed and may reduce unwanted side effects caused by constitutive activation of stress responses. One notable example of this type of strategy comes from a member of the Solanaceae with transgenic potato tubers that expressed the Arabidopsis AP2/ERF gene AtDREB1A under the control of the drought stress-inducible *RD29A* promoter or the strong constitutive *CaMV35S* promoter [26]. Metabolite profiling of the transgenic lines revealed elevated levels of glutathione and GABA in addition to β-cyanoalanine, which is a biosynthesis by product of ethylene. Both glutathione and GABA are components of what we have described as the plant core metabolite response to drought stress and it appears that use of transcription factors similar to AtDREB1A (such as NtERF218 and NtERF228) as a transgene holds promise, especially in conjunction with inducible promoters such as the *NtWRKY69* promoter (Figure 9).

Systems of reduced complexity have served plant scientists well in the discovery of many important components of the responses of plants to different abiotic stresses. Our data have likewise revealed new potential targets for crop improvement (Table 1). However, we are well aware that field conditions are much more complex that these hydroponics/growth room based studies and that our discoveries will need to be extended to field conditions. For example, studies have revealed that the response of plants to a combination of two different abiotic stresses is unique and cannot be directly extrapolated from the response of plants to each of the different stresses applied individually [41]. Drought tolerance is a complex quantitative and multigenic trait with a significant environmental component [37,40]. As a result, the genetic control of traits associated with tolerance to drought often shows low heritability. Therefore, one of the major hurdles in using the toolbox of genes, promoters and metabolites described in Table 1 to improve drought responses in Solanaceae crops lies not in producing these plants but in accurately defining them. The ongoing development of new phenomics technologies promises to greatly facilitate the characterization of transgenic lines, especially under field conditions. For this reason, field phenotyping systems may give us our best opportunity to determine whether strategies to improve drought stress responses can actually produce plants with increased drought tolerance.

## Conclusions

We propose components of a core metabolic response to drought stress in plants but also show that major responses to drought stress at the metabolome and transcriptome levels are family specific.

## Methods

### Plant materials, growth conditions and drought stress treatments

Tobacco cv. ‘Burley 21’ seedlings were germinated on agar plates. When the plants had grown to almost reach the top of the petri dish, they were then transferred to a mini hydroponics set up in sterile MK-5 polycarbonate vessels with half-strength MS liquid medium. Plants were supported by plastic inserts with holes in them for the plants and grown for 2 weeks at 25°C. They were then subjected to dehydration in a growth room for 0 (control), 20, 40, 60, 120 and 240 minutes by removing them from the liquid using the supports. Each time point consisted of three replicates with 20 plants per replicate. Roots and leaves were harvested by flash freezing in liquid nitrogen. All samples from the time course of water stress were taken during the morning.

Soybean W-82 seeds were grown in hydroponics using 0.5 × Hoagland’s solution, pH 5.8 in a growth chamber with a 16 hour/8 hour day/night cycle at 25°C and 50% relative humidity. After 30 days, plants were subjected to drought stress for 30 minutes, one hour, two hours, three hours and five hours by removing them by means of the plastic lid supports without touching the plants. Leaves and roots were harvested by flash freezing in liquid nitrogen. Nine plants were utilized for each time-point (three replicates per time-point and three plants per replicate).

### Osmotic potential, Stomatal conductance, and relative water content

A Vapro 5520 osmometer (Wescor, Logan, UT, USA) was used to measure the osmotic potential using 10 μL cell sap samples. Stomatal conductance was determined using an SC-1 leaf porometer (Decagon, Pullman, WA, USA). Relative water content was measured following the protocol of Gonzalez and Gonzalez-Vilar [21].

### Total RNA extraction

RNA isolation was performed using an RNeasy kit (Qiagen, Valencia, CA, USA) following the manufacturer’s protocol. Genomic DNA contamination was removed using an Ambion DNA-free and DNase removal kit (Life Technologies, Carlsbad, CA, USA). 10 μg total RNA from each sample was used for micro-array analysis.

### Transcriptomics

A Nimblegen custom oligo array was used for transcriptome analyses. The oligo array contained 385,000 probes with approximately seven 60-mer probes for each gene sequence. The sequences came from three different sources. Firstly, 40,000 individual genomic survey sequence reads (GSSs) from the Tobacco Genome Initiative that had the highest E-value hits to proteins in the NCBI nr database (A data set of 1,159,022 genomic survey sequences was downloaded from the TGI http://solgenomics.net/ in 2008), secondly all TOBFAC transcription factors [52], and thirdly the Version 4.0 DFCI Tobacco Gene Index EST sequences (NTGI4; ftp://occams.dfci.harvard.edu/pub/bio/tgi/data/Nicotiana_tabacum/; file name: Source files/NTGI.071508.fasta). The genome survey sequences have subsequently been deposited at The National Center for Biotechnology Information http://www.ncbi.nlm.nih.gov/; file name: Source files/TGSS_expressed.fasta although these deposited sequences were the result of re-reading to extend the length of the sequences and have more errors. Oligoarray experiments were performed at MOgene LC (St Louis, MO) using their standard protocols. Data analysis was performed using ArrayStar v4. Differential expression was calculated using 90% confidence (FDR Benjamini Hochberg) and 8-fold change as the cut off. We chose a high fold inducibility because under the experimental conditions very high inducibilities were obtained and a cut off value of 4-fold gave several thousand genes at the later time points. 8-fold was chosen to focus on only the most highly induced or repressed genes. 90% confidence was chosen because at early time points some genes failed the 95% confidence limit but as the time course progressed, these and other genes typically passed the 95% or even the 99% confidence limit. Pathway visualization was performed using our tobacco MapMan mapping [39].

For soybean, a custom made 12 × plex array was designed by Roche NimbleGen, Inc. containing multiple 60mer oligomers to all high and low confidence genes from the GLYMAv1.0 release of the soybean genome. This was used for soybean expression analyses. Hybridizations and data analyses were performed in an identical way to tobacco.

### Metabolomics

The same set of tobacco samples that was used for transcriptomic analyses was also used for metabolomics analysis at Metabolon, Inc. (North Carolina) using their analysis pipeline. The global unbiased metabolic profiling platform was based on a combination of three independent platforms: UHLC/MS/MS2 optimized for basic species, UHLC/MS/MS2 optimized for acidic species, and GC/MS. This platform has been described in detail [16]. Compounds were identified by comparison to library entries of purified standards or recurrent unknown entities, a total of over one thousand compounds. Following log transformation and imputation with minimum observed values for each compound, Welch’s two-sample *t*-test was used to identify biochemicals that differed significantly between experimental groups. Biochemicals that achieved statistical significance (p ≤ 0.05), as well as those approaching significance (0.05 < p < 0.1), were highlighted in the data set. An estimate of the false discovery rate (q-value) was also calculated to take into account the multiple comparisons that normally occur in metabolomic-based studies. The q-value describes the false discovery rate; a low q-value (q < 0.10) is an indication of high confidence in a result.

### Phylogenetic analyses

The amino acid sequences of the DNA binding domains of WRKY, and AP2/ERF transcription factors, and the complete amino acid sequences of HDZIP transcription factors, were used to construct phylogenetic trees. Alignments were constructed using MUSCLE [15] and the following parameters; Gap Penalties: Gap open −2.9, Gap Extended 0, Hydrophobicity multiplier 1.2 Memory/Iterations: Max Memory in MB 4095, Max Iterations 8; Clustering Method Iteration 1, 2 (UPGMB), Clustering Method (Other Iterations (UPGMB), Min. Diag. Length (Lambda) 24. Alignments are presented as Additional file 12: Table S8, Additional file 13: Table S9, Additional file 14: Table S10. In each case, the evolutionary history was inferred using the Neighbor-Joining method [55]. The percentage of replicate trees in which the associated taxa clustered together in the bootstrap test (1000 replicates) were determined [17]. The evolutionary distances were computed using the Poisson correction method [73] and are in the units of the number of amino acid substitutions per site. All ambiguous positions were removed for each sequence pair. Evolutionary analyses were conducted in MEGA6 [62]. All positions containing alignment gaps and missing data were eliminated in pairwise sequence. The amino acid sequences of the DNA binding domains were taken from the TOBFAC database (tobacco), TAIR (Arabidopsis), Phytozome (soybean), and solgenomics.net (tomato). Expression data came from tobacco (GEO accession GSE67434), soybean (GEO accession GSE49537), Arabidopsis [33] and tomato [20,25,38]. Data on Arabidopsis gene function were taken from the TAIR website (http://www.arabidopsis.org.).

### Promoter analyses

The choice of promoters was dictated by available promoter sequence. The blast search and contig builder functions in the TOBFAC data base were used to extend genomic sequences to include the promoters. Where promoter sequences were obtained, the promoter regions were PCR amplified, verified by sequencing and inserted as HindIII-SacI fragments the pGPTV-GUS-KAN [2,53] or pGPTV-GFP-KAN binary vectors. Tobacco transformation was performed following the protocol of Gallois and Marinho [18]. Four-week old seedlings were subjected to different treatments. Dehydration was imposed by removing the plants from liquid medium into an empty MK-5 polyethylene vessel. The vessels with the plants were then placed into a large plastic container with water to allow for slow dehydration of the plant. Wounding was achieved by pressure exerted by serrated tweezers. Cold treatments were performed by transferring the plants to 4°C. GFP activity was monitored at 5, 12 and 24 h while GUS activity was observed after 24 h. Histochemical staining of GUS activity was performed as described by Jefferson et al. [27]. GFP visualization of transgenic plants was performed using an Olympus SZX16 Epi-Fluorescent stereo microscope. The promoter sequences of NtWRKY69, NtWRKY70, NtWRKY3, UPLL2, and galactinol synthase are available under the GenBank accession numbers x-x.

### Protein motif analysis

Analysis of selected full length AP2/ERF transcription factors for protein domain architecture and putative conserved domains was carried out using MEME (http://meme.sdsc.edu/meme/cgi-bin/meme.cgi). The settings were; any number of repetitions of a single motif, minimum width of a motif ten amino acids, maximum width of a motif sixty amino acids, maximum number of motifs to find nine.

### Availability of supporting data

The tobacco oligo array data is available at the Gene Expression Omnibus online repository (NCBI GEO) as GEO accession GSE67434.

The soybean oligo array data is available at the Gene Expression Omnibus online repository (NCBI GEO) as GEO accession GSE49537 (http://www.ncbi.nlm.nih.gov/geo/query/acc.cgi?token=xdcnpsawooaywbs&acc=GSE49537).

## Additional files

Additional file 1: Table S1. Changes in metabolites in tobacco root and leaf tissue during drought. Additional file 2: Figure S1. Principal Component Analysis (PCA) plots of all independent data points. Additional file 3: Table S2. Genomic survey sequence reads from the Tobacco Genome Initiative project and TOBFAC transcription factors present on the oligo array together with their identification numbers on the oligo array. Additional file 4: Table S3. Version 3.0 DFCI Tobacco Gene Index EST sequences that were present on the oligo array. Additional file 5: Table S4. Genes that are up- or down-regulated at least 8-fold during drought. Additional file 6: Figure S2. Roles for the plant hormones ABA, JA, and ethylene in the response to drought stress in tobacco. Additional file 7: Table S5. Early and late up-regulated transcription factor genes during drought. Additional file 8: Figure S3. MapMan visualization of changes in transcription factor mRNA levels during drought stress in tobacco. Additional file 9: Table S6. The WRKY transcription factor family in soybean together with apparent pseudogenes. Additional file 10: Figure S4. Protein architecture of selected Group IX AP2/ERF transcription factors as determined by MEME. Additional file 11: Table S7. Drought inducibility of Subgroup 3 Group IX ERF transcription factor genes from tobacco and soybean. Additional file 12: Table S8. MUSCLE alignment used by MEGA6 to create the Neighbor-Joining phylogenetic tree of the WRKY family. Additional file 13: Table S9. MUSCLE alignment used by MEGA6 to create the Neighbor-Joining phylogenetic tree of the AP2/ERF family. Additional file 14: Table S10. MUSCLE alignment used by MEGA6 to create the Neighbor-Joining phylogenetic tree of part of the homeodomain family.
