# Supplementary figures and images for: Tobacco drought stress responses reveal new targets for Solanaceae crop improvement
Source: BMC Genomics. 2015 Jun 30;16(1):484. doi: 10.1186/s12864-015-1575-4 (PMC4485875; doi:10.1186/s12864-015-1575-4)

## LEAF

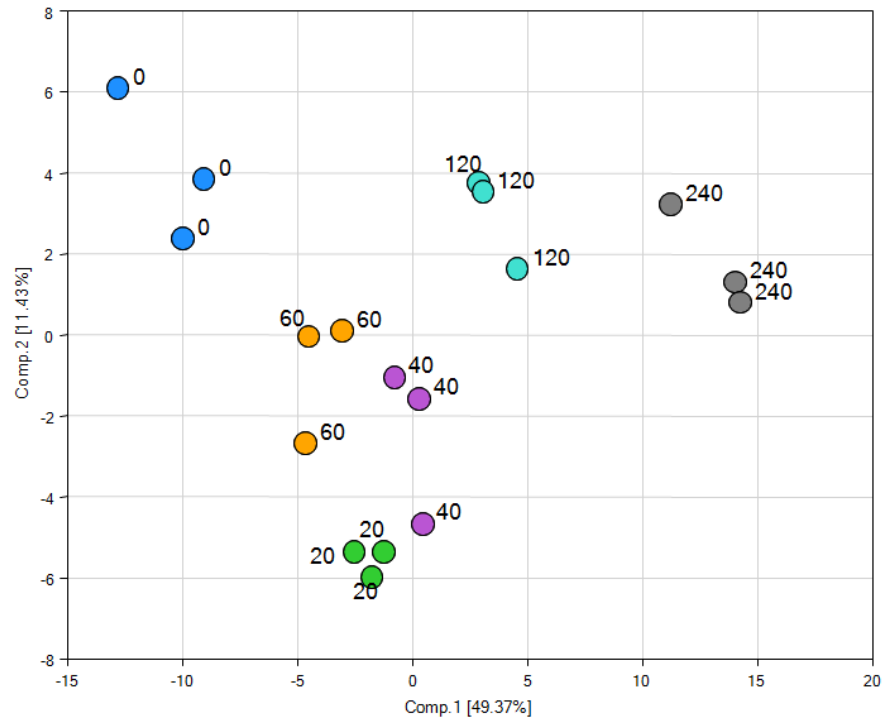

## ROOT

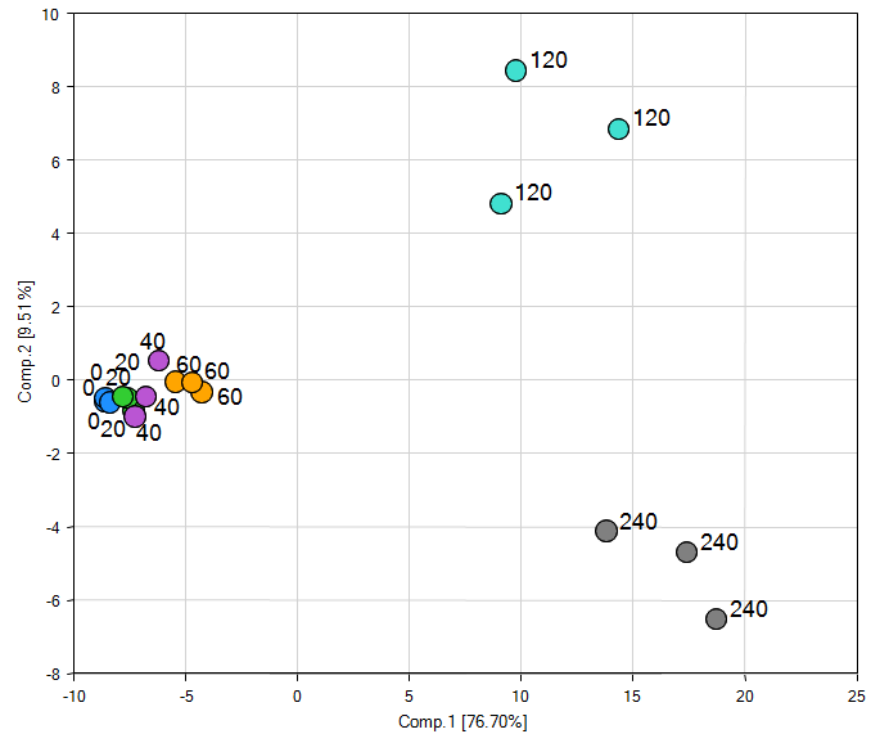

Supplement: Additional file 2: Figure S1. — Principal Component Analysis (PCA) plots of all independent data points. [file 12864_2015_1575_MOESM2_ESM.pdf]

**A.**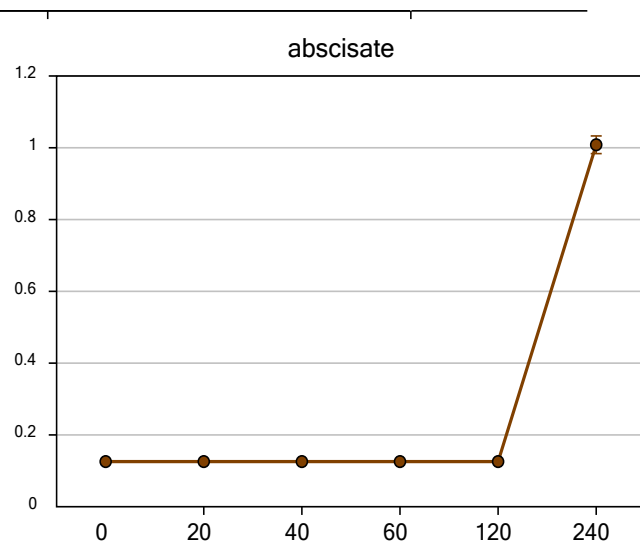**B.**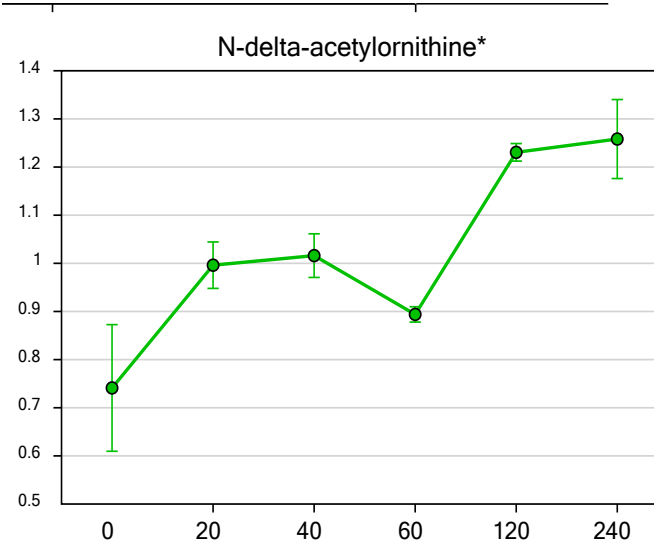**C.**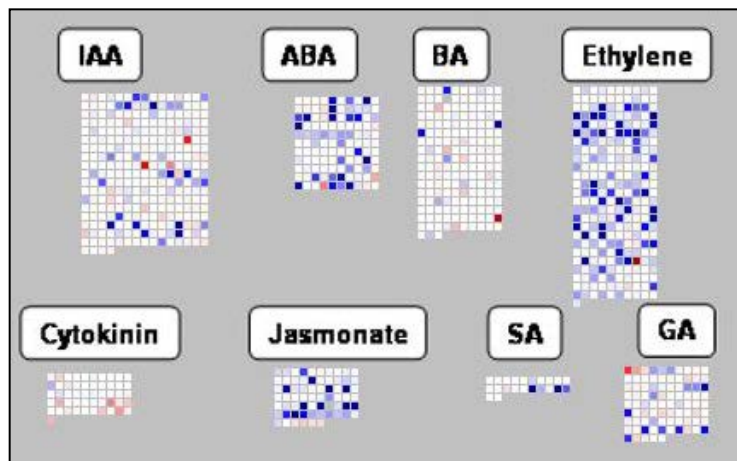**D.**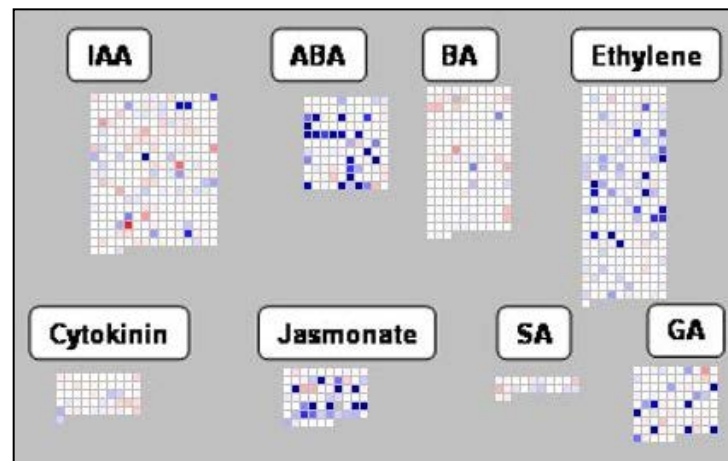

E.

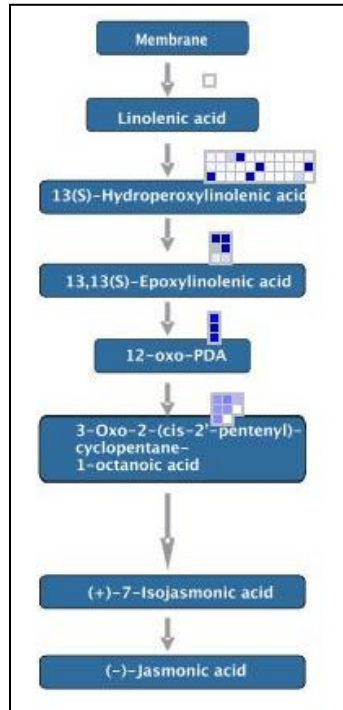

F.

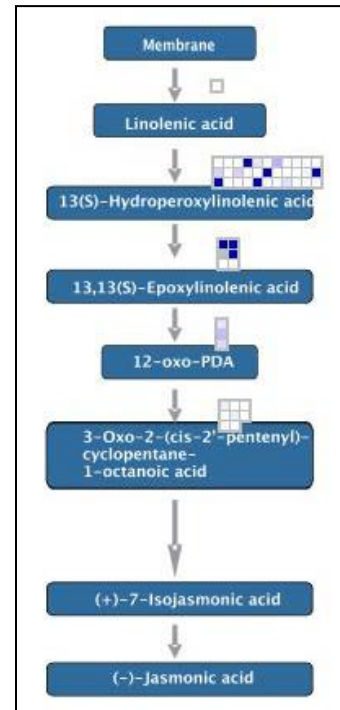

Supplement: Additional file 6: Figure S2. — Roles for the plant hormones ABA, JA, and ethylene in the response to drought stress in tobacco. [file 12864_2015_1575_MOESM6_ESM.pdf]
