# Supplementary material for: Tobacco drought stress responses reveal new targets for Solanaceae crop improvement
Source: BMC Genomics. 2015 Jun 30;16(1):484. doi: 10.1186/s12864-015-1575-4 (PMC4485875; doi:10.1186/s12864-015-1575-4)
Supplement: Additional file 3: Table S2. — Genomic survey sequence reads from the Tobacco Genome Initiative project and TOBFAC transcription factors present on the oligo array together with their identification numbers on the oligo array. [file 12864_2015_1575_MOESM3_ESM.docx]

**Additional_file_3 – as DOCX**

Additional file 3: Table S2.

Genomic survey sequence reads from the Tobacco Genome Initiative project and TOBFAC transcription factors present on the oligo array together with their identification numbers on the oligo array.

This file is over the 20MB file limit and so this is merely a place holder
