# Supplementary material for: Tobacco drought stress responses reveal new targets for Solanaceae crop improvement
Source: BMC Genomics. 2015 Jun 30;16(1):484. doi: 10.1186/s12864-015-1575-4 (PMC4485875; doi:10.1186/s12864-015-1575-4)
Supplement: Additional file 4: Table S3. — Version 3.0 DFCI Tobacco Gene Index EST sequences that were present on the oligo array. [file 12864_2015_1575_MOESM4_ESM.docx]

**Additional_file_4 – as DOCX**

Additional file 4: Table S3.

Version 3.0 DFCI Tobacco Gene Index EST sequences that were present on the oligo array.

This file is over the 20MB file limit and so this is merely a place holder
