# Supplementary material for: Tobacco drought stress responses reveal new targets for Solanaceae crop improvement
Source: BMC Genomics. 2015 Jun 30;16(1):484. doi: 10.1186/s12864-015-1575-4 (PMC4485875; doi:10.1186/s12864-015-1575-4)

# a. Leaf 20 minutes

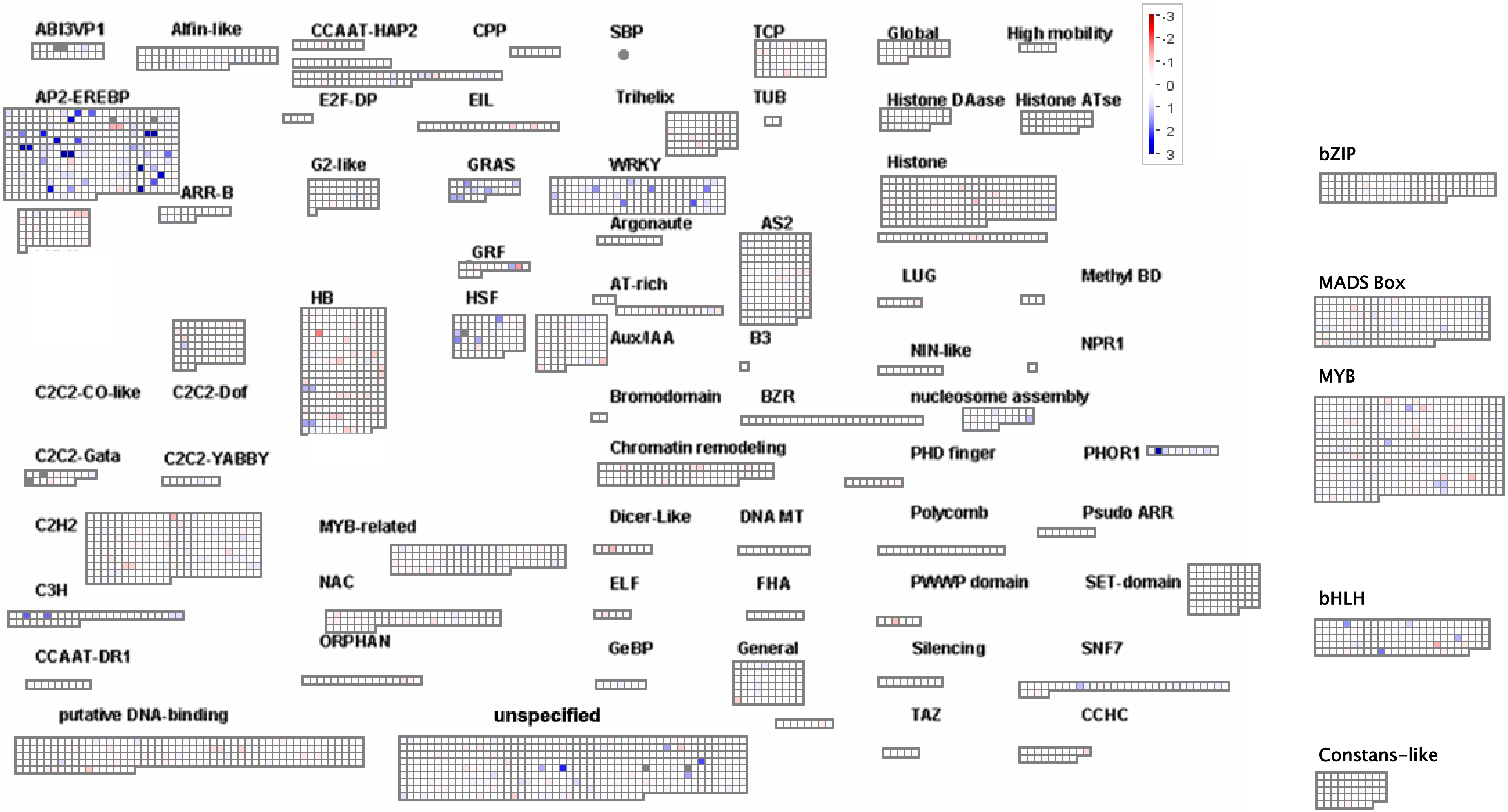

## b. Leaf 40 minutes

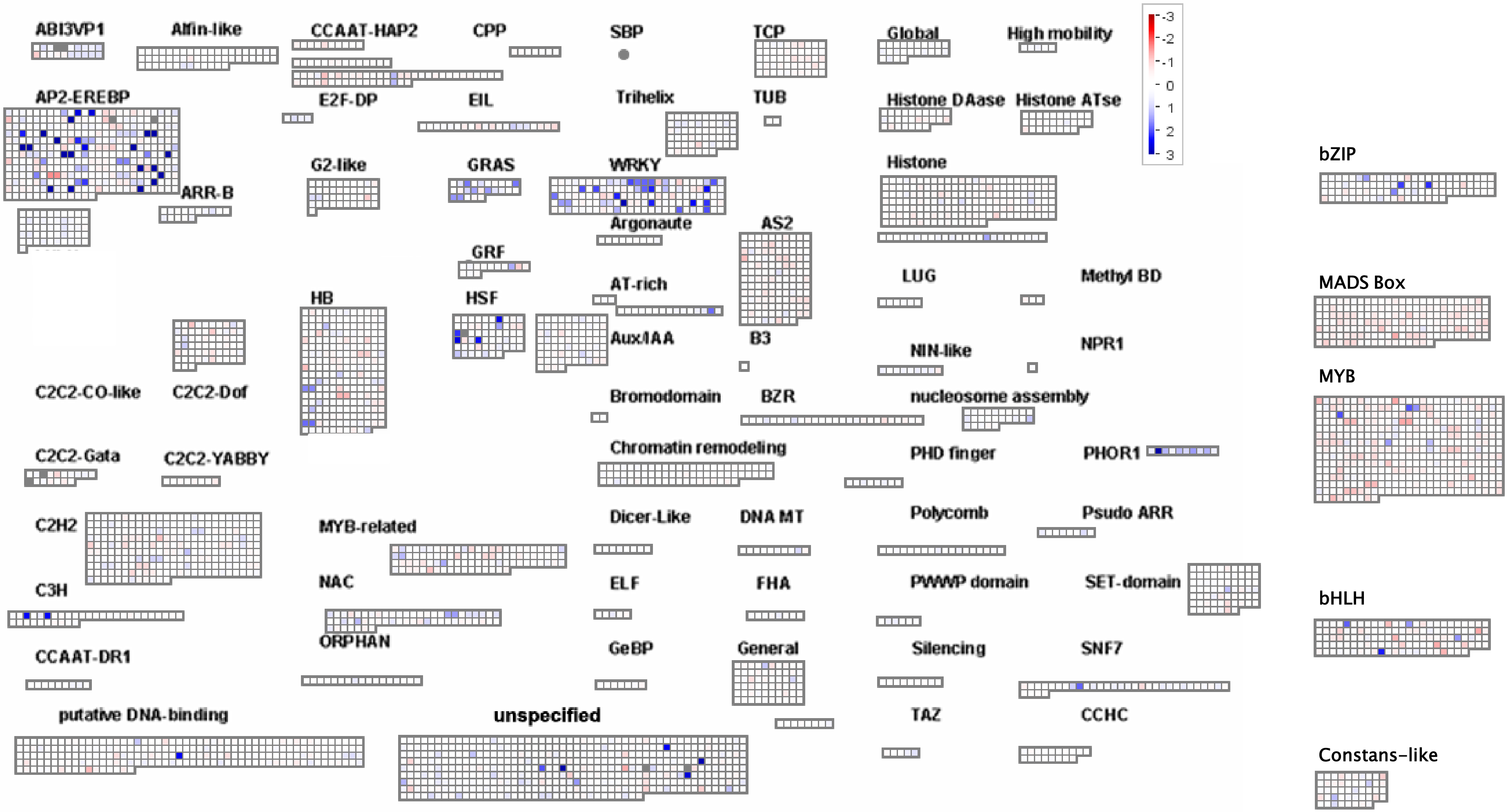

## c. Leaf 1 hour

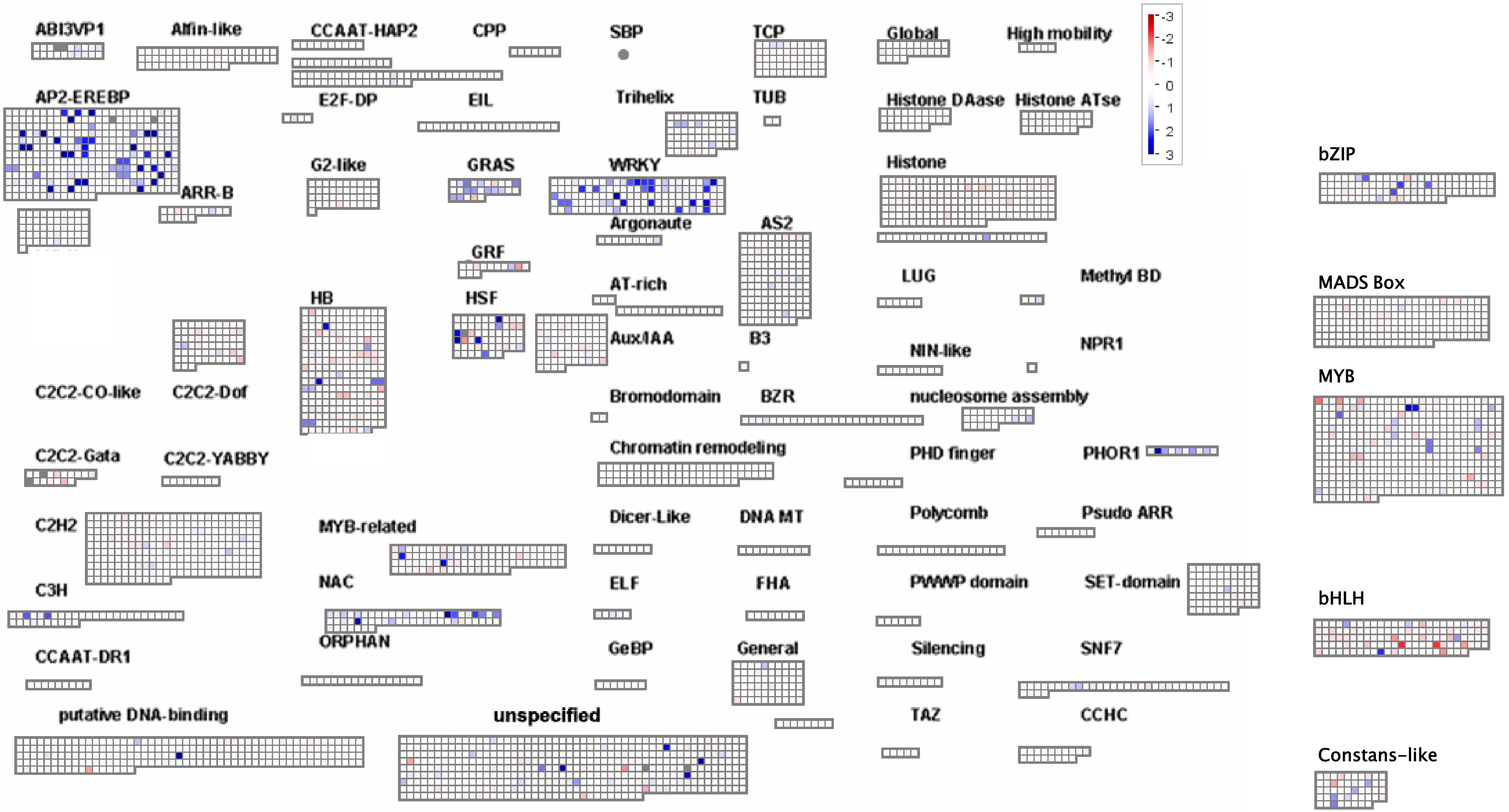
ABI3VP1
Alfin-like
CCAAT-HAP2
CPP
SBP
TCP
Global
High mobility
AP2-EREBP
E2F-DP
EIL
Trihelix
TUB
Histone DAase
Histone ATse
ARR-B
G2-like
GRAS
WRKY
Histone
Argonaute
AS2
GRF
LUG
Methyl BD
AT-rich
HB
HSF
Aux/IAA
B3
NIN-like
NPR1
C2C2-CO-like
C2C2-Dof
Bromodomain
BZR
nucleosome assembly
Chromatin remodeling
PHD finger
PHOR1
C2C2-Gata
C2C2-YABBY
C2H2
MYB-related
Dicer-Like
DNA MT
Polycomb
Psudo ARR
C3H
NAC
ELF
FHA
PWWP domain
SET-domain
ORPHAN
CCAAT-DR1
GeBP
General
Silencing
SNF7
putative DNA-binding
unspecified
TAZ
CCHC
-3
-2
-1
0
1
2
3
bZIP
MADS Box
MYB
bHLH
Constans-like

# d. Leaf 2 hours

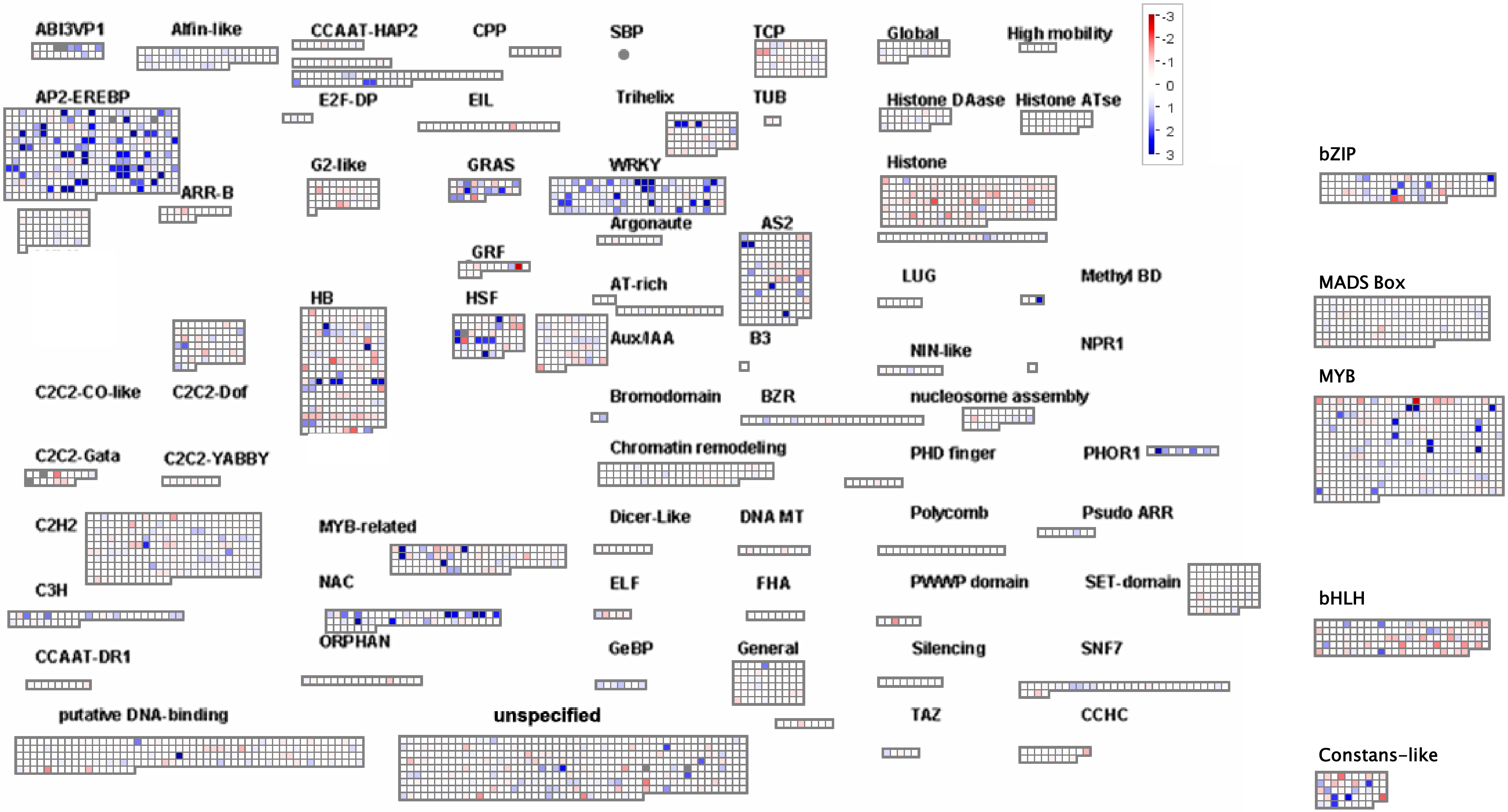

# e. Leaf 4 hours

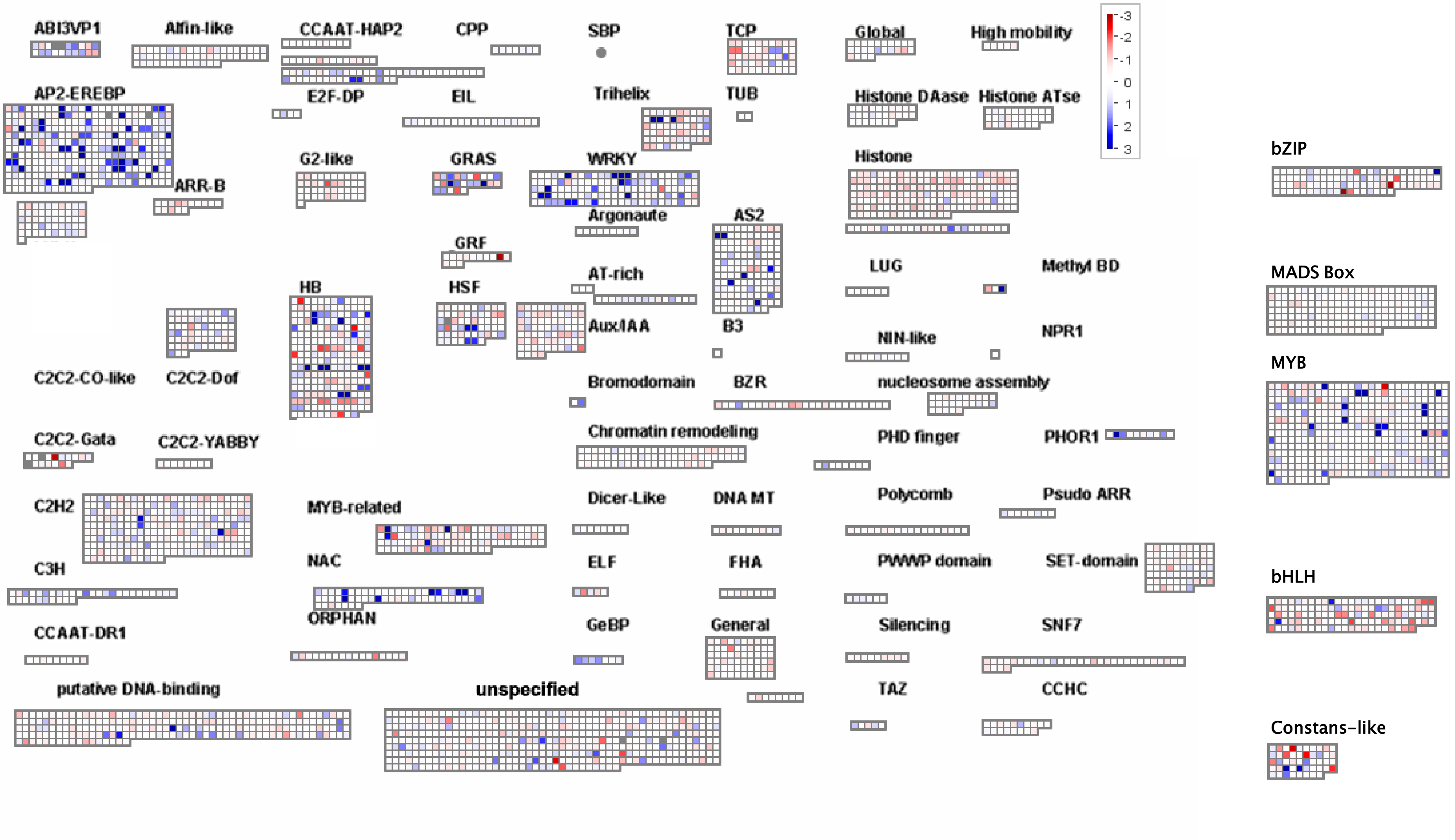

# f. Root 20 minutes

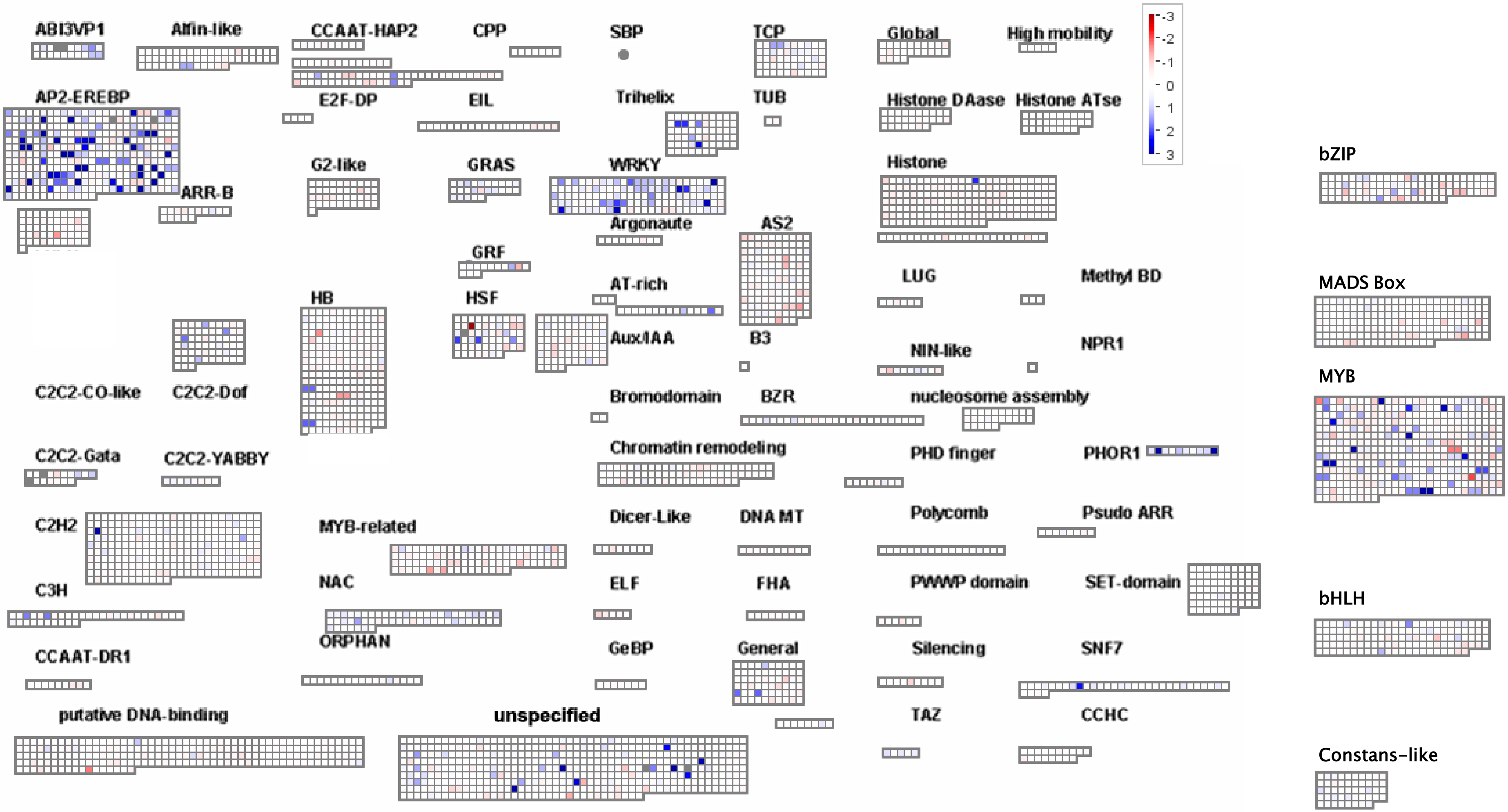

## g. Root 40 minutes

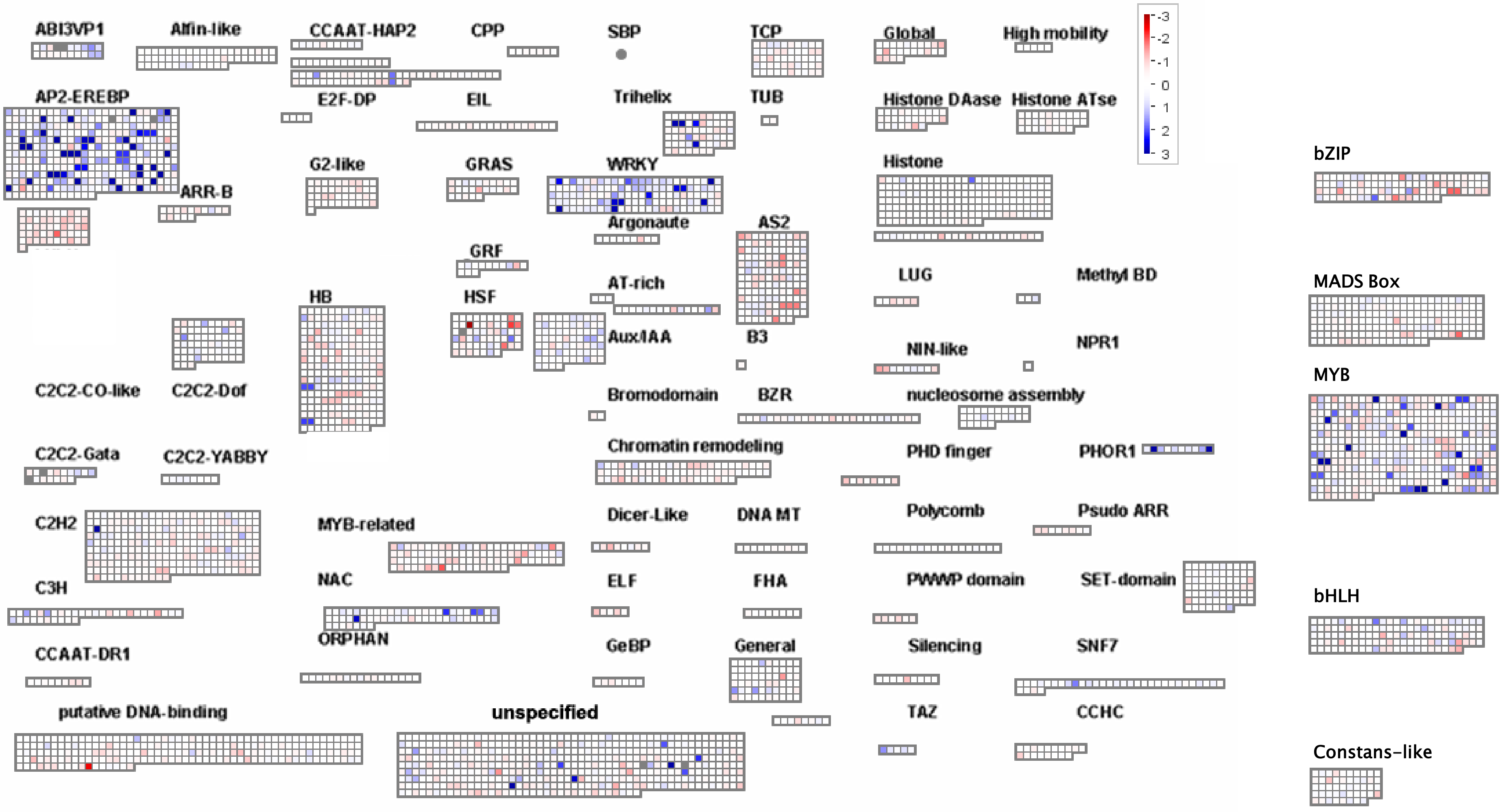

# h. Root 1 hour

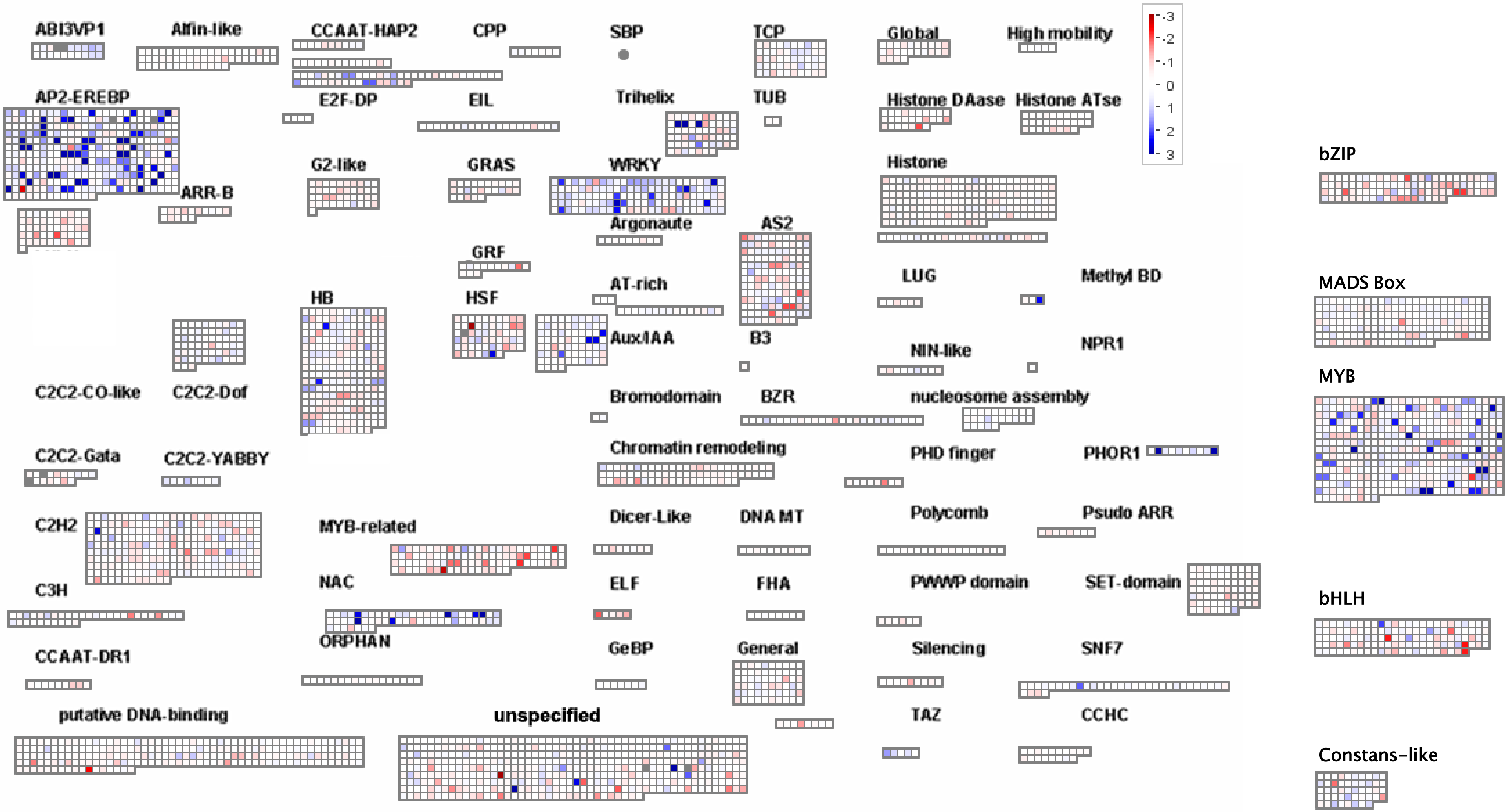
ABI3VP1
Alfin-like
CCAAT-HAP2
CPP
SBP
TCP
Global
High mobility
-3
-2
-1
0
1
2
3
AP2-EREBP
E2F-DP
EIL
Trihelix
TUB
Histone DAase
Histone ATse
bZIP
ARR-B
G2-like
GRAS
WRKY
Histone
Argonaute
AS2
GRF
LUG
Methyl BD
MADS Box
AT-rich
HB
HSF
Aux/IAA
B3
NIN-like
NPR1
MYB
C2C2-CO-like
C2C2-Dof
Bromodomain
BZR
nucleosome assembly
C2C2-Gata
C2C2-YABBY
Chromatin remodeling
PHD finger
PHOR1
C2H2
MYB-related
Dicer-Like
DNA MT
Polycomb
Psudo ARR
C3H
NAC
ELF
FHA
PWWP domain
SET-domain
bHLH
ORPHAN
CCAAT-DR1
GeBP
General
Silencing
SNF7
putative DNA-binding
unspecified
TAZ
CCHC
Constans-like

# i. Root 2 hours

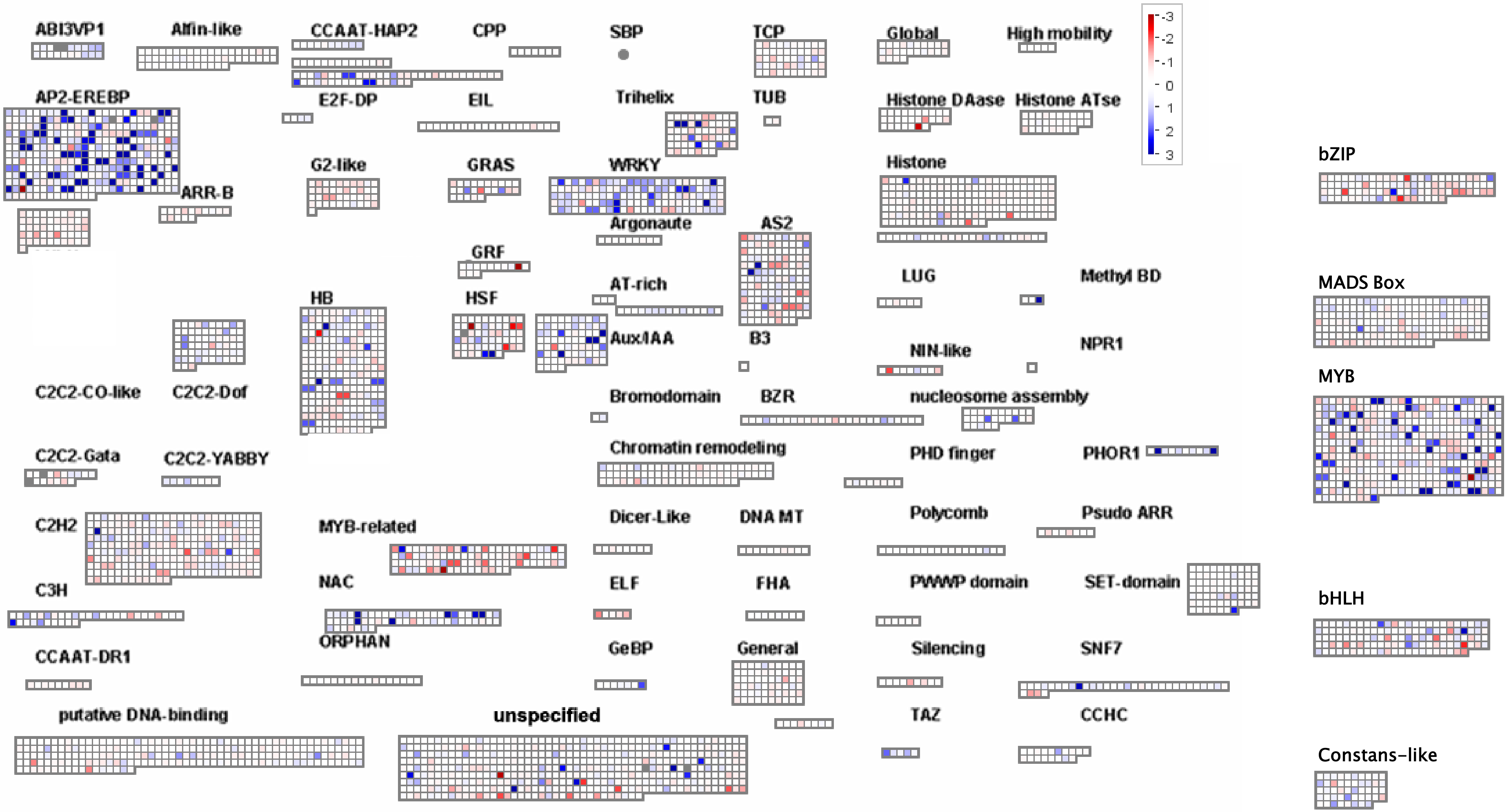

## j. Root 4 hours

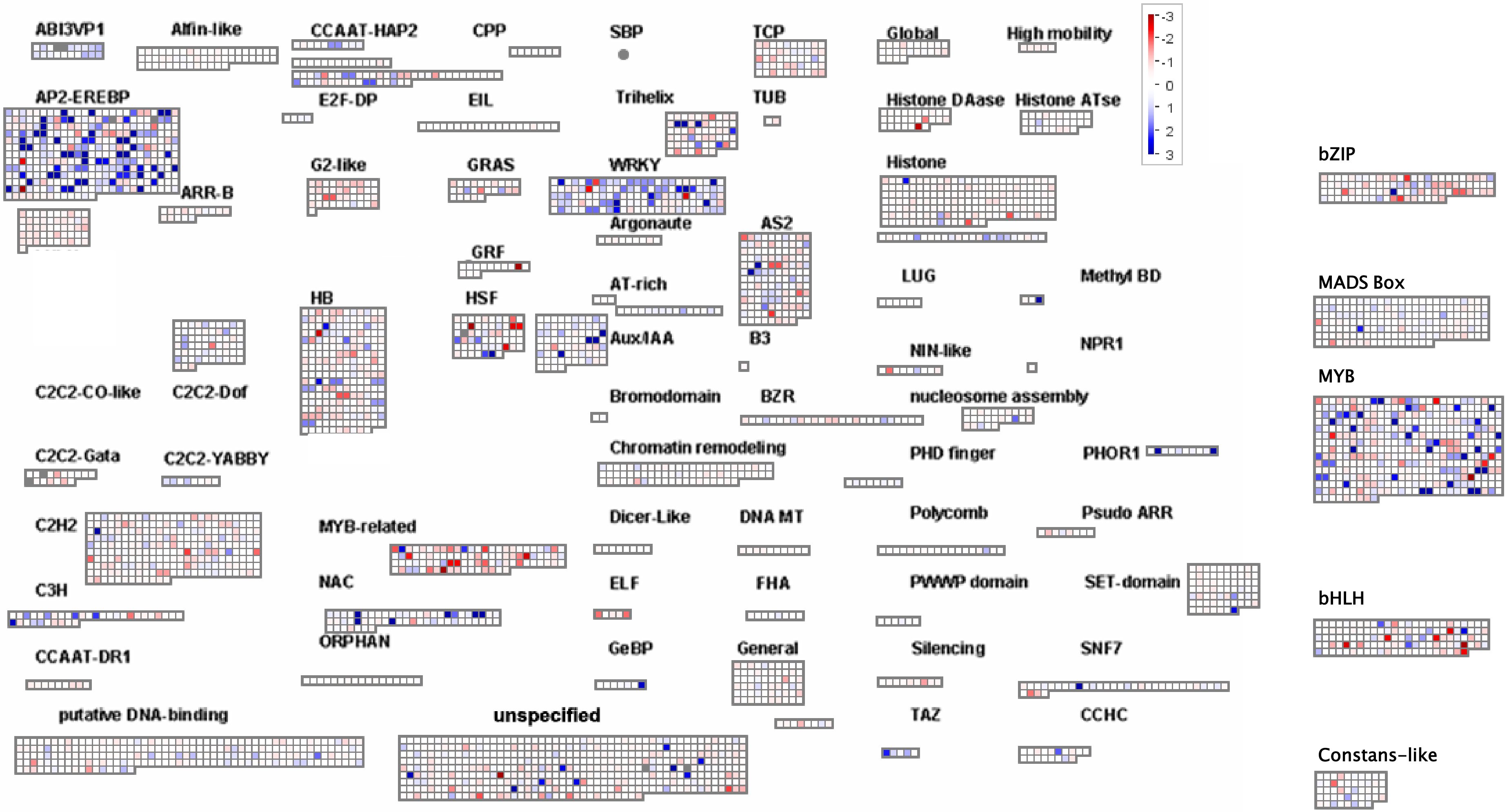

Supplement: Additional file 8: Figure S3. — MapMan visualization of changes in transcription factor mRNA levels during drought stress in tobacco. [file 12864_2015_1575_MOESM8_ESM.pdf]
