# Supplementary material for: Tobacco drought stress responses reveal new targets for Solanaceae crop improvement
Source: BMC Genomics. 2015 Jun 30;16(1):484. doi: 10.1186/s12864-015-1575-4 (PMC4485875; doi:10.1186/s12864-015-1575-4)
Supplement: Additional file 9: Table S6. — The WRKY transcription factor family in soybean together with apparent pseudogenes. [file 12864_2015_1575_MOESM9_ESM.doc]

| **Gene Name** | **Position** | **Gene model** | **Group** | **Comments** | **Structure** |
| --- | --- | --- | --- | --- | --- |
|  |  |  |  |  |  |
| ***WRKY1*** | Gm10:36663345..36667781 | Glyma10g27860 incorrect | IIb | Published EU019552 partial | Published EU019552 partial |
| ***WRKY1/7*** | Gm14:1152672..1158224 | Glyma14g01980 | I | Published AY323128 | Published AY323128 |
| ***WRKY2*** | Gm10:45452410..45454003 | Glyma10g37460 | IIe | Published EU019553 partial | Published EU019553 partial |
| ***WRKY3*** | Gm02:50581035..50586821 | Glyma02g46690 | I | Published PTGm01590.1 partial | Published PTGm01590.1 partial |
| ***WRKY4*** | Gm08:20589336..20594044 | Glyma08g26230 | I | Published EU375355 | Published EU375355 |
| ***WRKY5*** | Gm01:43157312..43160088 | Glyma01g31920 | I | Published EU019554 partial | Published EU019554 partial |
| ***WRKY6*** | Gm08:10932587..10935883 | Glyma08g15050 | IIc | Published DQ322690 | Published DQ322690 |
| ***WRKY7*** | Gm16:33345971..33347226 | Glyma16g29560 incorrect | IIe | Glyma16g29500 is almost direct duplication | Glyma16g29500 is almost direct duplication |
| ***WRKY8*** | Gm19:46863575..46865461 | Glyma19g40470 | IIe | Published EU019556 partial | Published EU019556 partial |
| ***WRKY9*** | Gm01:6797680..6802906 | Glyma01g06550 | I | Published EU019557 partial | Published EU019557 partial |
| ***WRKY10*** | Gm18:56995547..56997166 | Glyma18g47300 | IIe | Published EU375344 incorrect | Published EU375344 incorrect |
| ***WRKY11*** | Gm05:24873227..24875361 | Glyma05g20710 | IId | Published EU375356 | Published EU375356 |
| ***WRKY12*** | Gm01:54434358..54435804 | Glyma01g43420 | III | Published EU019558 partial | Published EU019558 partial |
| ***WRKY13*** | Gm13:124483..126098 | Glyma13g00380 | IId | Published DQ322694 | Published DQ322694 |
| ***WRKY14*** | Gm11:3985100..3986762 | Glyma11g05650 | IId | Published EU019559 partial | Published EU019559 partial |
| ***WRKY15*** | Gm03:39497541..39499620 | Glyma03g31630 | IId | Published EU019560 partial | Published EU019560 partial |
| ***WRKY16*** | Gm12:37176018..37178776 | Glyma12g33990 | IIe | Published EU375351 | Published EU375351 |
| ***WRKY17*** | Gm06:4658765..4660532 | Glyma06g06530 | IIa | Published EU019561 partial | Published EU019561 partial |
| ***WRKY18*** | Gm08:43556504..43562484 | Glyma08g43770 | I | Published EU019562 partial | Published EU019562 partial |
| ***WRKY19*** | Gm11:30493000..30496843 | Glyma11g29720 | I | Published EU019563 partial | Published EU019563 partial |
| ***WRKY20*** | Gm05:40700855..40703837 | Glyma05g36970 | III | Published EU019564 is a possible chimeric of Glyma08g02580 and Glyma05g36970 |  |
| ***WRKY21*** | Gm04:45842918..45845805 | Glyma04g39650 | IIc | Published DQ322691 | Published DQ322691 |
| ***WRKY22*** | Gm02:14383111..14385983 | Glyma02g15920 | IId | Published EU375352 | Published EU375352 |
| ***WRKY23*** | Gm09:442505..444978 | Glyma09g00820 | IIb | Published EU019565 partial | Published EU019565 partial |
| ***WRKY24*** | Gm17:6042363..6050508 | Glyma17g08170 | I | Published EU019566 partial | Published EU019566 partial |
| ***WRKY25*** | Gm08:898817..900025 | Glyma08g01430 | IIc | Published EU019567 | Published EU019567 |
| ***WRKY26*** | Gm09:2825485..2827480 | Glyma09g03900 | IIc | Published EU019568 partial | Published EU019568 partial |
| ***WRKY27*** | Gm15:289709..291752 | Glyma15g00570 | IIa | Published DQ322695 | Published DQ322695 |
| ***WRKY28*** | Gm01:7343379..7347036 | Glyma01g06870 | IIc | Published EU019572 is possibly a chimeric of Glyma02g12830 and Glyma01g06870 | Published EU019572 is possibly a chimeric of Glyma02g12830 and Glyma01g06870 |
| ***WRKY29*** | Gm03:40963198..40966641 | Glyma03g33380 | I ? | Published EU019569 partial  Unusual N terminal domain | Published EU019569 partial |
| ***WRKY30*** | Gm17:37802115..37803840 | Glyma17g33890 incorrect | IIa | Published EU019570 partial | Published EU019570 partial |
| ***WRKY31*** | Gm14:19599904..19602834 | Glyma14g17730 | IId | Published EU019571 partial | Published EU019571 partial |
| ***WRKY32*** | Gm02:11099620..11106899 | Glyma02g12830 | IIc | Published EU019572 is possibly a chimeric of Glyma02g12830 and Glyma01g06870 | Published EU019572 is possibly a chimeric of Glyma02g12830 and Glyma01g06870 |
| ***WRKY33*** | Gm17:4598267..4599927 | Glyma17g06450 | IId | Published DQ322696 partial | Published DQ322696 partial |
| ***WRKY34*** | Gm07:43769034..43772340 | Glyma07g39250 | IIb | Published EU019573 partial | Published EU019573 partial |
| ***WRKY35*** | Gm08:9144564..9146502 | Glyma08g12460 | III | - | - |
| ***WRKY36*** | Gm13:39385564..39388414 | Glyma13g38630 | IIb | Published EU019575 partial | Published EU019575 partial |
| ***WRKY37*** | Gm06:5977428..5979246 | Glyma06g08120 | IId | Published EU375346 | Published EU375346 |
| ***WRKY38*** | Gm18:57870036..57870165 | Glyma18g48460 incorrect | IIc | Published EU375347. | Published EU375347. |
| ***WRKY39*** | Gm02:45077216..45080591 | Glyma02g39870 | I | Published EU019576 partial | Published EU019576 partial |
| ***WRKY40*** | Gm08:11030907..11033261 | Glyma08g15210 | IIc | Published DQ322692 | Published DQ322692 |
| ***WRKY41*** | Gm03:44338324..44340268 | Glyma03g37870 | IIe | Published EU019577 partial | Published EU019577 partial |
| ***WRKY42*** | Gm15:14920606..14922626 | Glyma15g18250 | IId | Published EU019578 partial | Published EU019578 partial |
| ***WRKY43*** | Gm03:47155311..47157888 | Glyma03g41750 | III | Published EU019579 | Published EU019579 |
| ***WRKY44*** | Gm12:26693632..26702804 | Glyma12g23950 | I | Published EU019580 partial | Published EU019580 partial |
| ***WRKY45*** | Gm04:45815356..45817894 | Glyma04g39620 incorrect | IIc | Published EU019581 partial | Published EU019581 partial |
| ***WRKY46*** | Gm08:1759380..1761603 | Glyma08g02580 | III | Published EU019582 partial | Published EU019582 partial |
| ***WRKY47*** | Gm09:5816998..5819128 | Glyma09g06980 | IId | Published EU019583 partial | Published EU019583 partial |
| ***WRKY48*** | Gm05:34988314..34990137 | Glyma05g29310 | IIe | Similar to EU019584 but not identical | Similar to EU019584 but not identical |
| ***WRKY49*** | Gm14:47231606..47234594 | Glyma14g38010 | I | Published EU019585 partial | Published EU019585 partial |
| ***WRKY50*** | Gm04:6305467..6307509 | Glyma04g08060 | IId | Published EU019586 | Published EU019586 |
| ***WRKY51*** | Gm03:44408648..44410605 | Glyma03g37940 | IIc | Published DQ322697 | Published DQ322697 |
| ***WRKY52*** | Gm13:37784670..37787467 | Glyma13g36540 | IIe | Published EU019587 | Published EU019587 |
| ***WRKY53*** | Gm19:33118636..33121087 | Glyma19g26400 | IIc | Published DQ322693 | Published DQ322693 |
| ***WRKY54*** | Gm10:1071000..1073128 | Glyma10g01450 | IIc | Published DQ322698 | Published DQ322698 |
| ***WRKY55*** | Gm07:5052692..5055589 | Glyma07g06320 | III | Published EU019588 partial | Published EU019588 partial |
| ***WRKY56*** | Gm08:17848920..17851229 | Glyma08g23380 | IIa | Published EU375348 | Published EU375348 |
| ***WRKY57*** | Gm18:54287774..54289782 | Glyma18g44560 | III | Published EU375353 | Published EU375353 |
| ***WRKY58*** | Gm04:46258589..46261143 | Glyma04g40130 | III | Published EU375354 | Published EU375354 |
| ***WRKY59*** | Gm06:50269204..50272918 | Glyma06g47880 incorrect | I | Published EU019589 partial | Published EU019589 partial |
| ***WRKY60*** | Gm16:2538841..2542084 | Glyma16g02960 | III | Published EU375357 | Published EU375357 |
| ***WRKY61*** | Gm06:11960949..11964356 | Glyma06g15220 | IIc | Published EU019590 | Published EU019590 |
| ***WRKY62*** | Gm18:4933285..4943284 | Glyma18g06360 incorrect | I | Published EU019591 partial | Published EU019591 partial |
| ***WRKY63*** | Gm17:37829359..37831606 | Glyma17g33920 | IIa | Published EU375349 but not identical | Published EU375349 but not identical |
| ***WRKY64*** | Gm18:57020541..57022120 | Glyma18g47350 | IIc | Published EU019592 partial | Published EU019592 partial |
| ***WRKY65*** | Gm02:51172240..51175230 | Glyma02g47650 | I | Published PTGm01579.1 partial | Published PTGm01579.1 partial |
| ***WRKY66*** | Gm20:3214359..3218968 | Glyma20g03410 | I | Published PTGm01508.1 partial | Published PTGm01508.1 partial |
| ***WRKY67*** | Gm13:44120121..44122122 | Glyma13g44730 | IIa | Published PTGm01511.1 partial | Published PTGm01511.1 partial |
| ***WRKY68*** | Gm14:528987..532674 | Glyma14g01010 | I | Published PTGm01514.1 partial | Published PTGm01514.1 partial |
| ***WRKY69*** | Gm02:1024414..1026553 | Glyma02g01420 | IIc | Published PTGm01515.1 partial | Published PTGm01515.1 partial |
| ***WRKY70*** | Gm09:43460578..43466591 | Glyma09g37930 | IIc | Published PTGm01517.1 partial | Published PTGm01517.1 partial |
| ***WRKY71*** | Gm07:1770021..1772340 | Glyma07g02630 | IIa | Published PTGm01518.1 | Published PTGm01518.1 |
| ***WRKY72*** | Gm10:2862806..2865704 | Glyma10g03820 | IId | Published PTGm01523.1 partial | Published PTGm01523.1 partial |
| ***WRKY73*** | Gm19:47231255..47236036 | Glyma19g40950 | IIb | Published PTGm01546.1 partial | Published PTGm01546.1 partial |
| ***WRKY74*** | Gm09:45810360..45812341 | Glyma09g41050 | III | Published PTGm01532.1 partial | Published PTGm01532.1 partial |
| ***WRKY75*** | Gm19:2182603..2185509 | Glyma19g02440 | IIb | Published PTGm01534.1 partial | Published PTGm01534.1 partial |
| ***WRKY76*** | Gm06:11533467..11536200 | Glyma06g14720 | III | Published PTGm01535.1 partial | Published PTGm01535.1 partial |
| ***WRKY77*** | Gm05:31474801..31478397 | Glyma05g25330 incorrect | ? | Gene model misses N-terminus | Gene model misses N-terminus |
| ***WRKY78*** | Gm18:7810847..7815984 | Glyma18g09040 | I | Published PTGm01553.1 partial | Published PTGm01553.1 partial |
| ***WRKY79*** | Gm02:10771403..10776397 | Glyma02g12490 | I | Published PTGm01558.1 partial | Published PTGm01558.1 partial |
| ***WRKY80*** | Gm18:59146858..59153215 | Glyma18g49830 | I | Published PTGm01567.1 partial | Published PTGm01567.1 partial |
| ***WRKY81*** | Gm06:25506384..25512608 | Glyma06g27440 | I | Published PTGm01570.1 partial | Published PTGm01570.1 partial |
| ***WRKY82*** | Gm14:10467577..10470309 | Glyma14g11920 | IIa | Published PTGm01577.1 partial | Published PTGm01577.1 partial |
| ***WRKY83*** | Gm01:51532858..51534768 | Glyma01g39600 | IId | Published PTGm01580.1 partial | Published PTGm01580.1 partial |
| ***WRKY84*** | Gm02:779474..781301 | Glyma02g01030 incorrect | IIb | Gene model short at 5 prime end | Gene model short at 5 prime end |
| ***WRKY85*** | Gm19:46938209..46939733 | Glyma19g40560 | IIc | Published PTGm01591.1 partial | Published PTGm01591.1 partial |
| ***WRKY86*** | Gm13:21520492..21524005 | Glyma13g17800 | IIb | Published PTGm01592.1 partial | Published PTGm01592.1 partial |
| ***WRKY87*** | Gm18:53614753..53617760 | Glyma18g44030 | I | Published PTGm01593.1 partial | Published PTGm01593.1 partial |
| ***WRKY88*** | Gm09:44180498..44181992 | Glyma09g39000 | IIc | Published PTGm01596.1 partial | Published PTGm01596.1 partial |
| ***WRKY89*** | Gm06:49042621..49045812 | Glyma06g46420 | IIb | - | - |
| ***WRKY90*** | Gm03:44705517..44709700 | Glyma03g38360 | IIb | - | - |
| ***WRKY91*** | Gm16:5308573..5311368 | Glyma16g05880 | IIc | Published PTGm01604.1 | Published PTGm01604.1 |
| ***WRKY92*** | Gm17:15865536..15867577 | Glyma17g18480 | IId | Published PTGm01605.1 | Published PTGm01605.1 |
| ***WRKY93*** | Gm04:12180262..12184979 | Glyma04g12830 | I | Published PTGm01607.1 partial | Published PTGm01607.1 partial |
| ***WRKY94*** | Gm15:8605796..8608443 | Glyma15g11680 | IIb | Published PTGm01608.1 partial | Published PTGm01608.1 partial |
| ***WRKY95*** | Gm14:2102160..2104636 | Glyma14g03280 | IIc | - | - |
| ***WRKY96*** | Gm02:41991807..41999444 | Glyma02g36510 | I | - | - |
| ***WRKY97*** | Gm02:49752821..49755287 | Glyma02g45530 | IIc | - | - |
| ***WRKY98*** | Gm18:9201202..9203210 | Glyma18g10330 incorrect | IIb | - | - |
| ***WRKY99*** | Gm18:16517642..16520212 | Glyma18g16170 | IIb | - | - |
| ***WRKY100*** | Gm19:43469371..43477681 | Glyma19g36100 | I | - | - |
| ***WRKY101*** | Gm07:41980980..41983038 | Glyma07g36640 | IIc | - | - |
| ***WRKY102*** | Gm07:40535078..40536634 | Glyma07g35380 | I | Small protein for a Group I gene | Small protein for a Group I gene |
| ***WRKY103*** | Gm18:57313719..57317367 | Glyma18g47740 | I | - | - |
| ***WRKY104*** | Gm18:58523898..58526841 | Glyma18g49140 | IIb | - | - |
| ***WRKY105*** | Gm20:3693574..3694677 | Glyma20g03820 incorrect | IIb | - | - |
| ***WRKY106*** | Gm09:46340943..46343377 | Glyma09g41670 | I | Group I with N-terminal WIKY | Group I with N-terminal WIKY |
| ***WRKY107*** | Gm09:43919025..43920987 | Glyma09g38580 incorrect | I | - | - |
| ***WRKY108*** | Gm09:43034765..43037417 | Glyma09g37470 | IIb | - | - |
| ***WRKY109*** | Gm03:5504134..5506013 | Glyma03g05220 incorrect | I | FGENESH predicts a longer protein at N-terminus | FGENESH predicts a longer protein at N-terminus |
| ***WRKY110*** | Gm17:909933..912461 | Glyma17g01490 | IIb | - | - |
| ***WRKY111*** | Gm17:2603960..2607060 | Glyma17g03950 | IIc | - | - |
| ***WRKY112*** | Gm17:3142520..3147240 | Glyma17g04710 | IIb | - | - |
| ***WRKY113*** | Gm17:7978190..7980809 | Glyma17g10630 incorrect | IIb | Gene model missed N-terminal portion | Gene model missed N-terminal portion |
| ***WRKY114*** | Gm06:21458477..21460096 | Glyma06g23990 incorrect | IIa | WKKY. Gene model incomplete | WKKY. Gene model incomplete |
| ***WRKY115*** | Gm05:36852764..36855865 | Glyma05g31800 | IIc | - | - |
| ***WRKY116*** | Gm07:15750032..15752879 | Glyma07g16040 incorrect | ? | Gene model truncated at both ends | Gene model truncated at both ends |
| ***WRKY117*** | Gm17:38083100..38084578 | Glyma17g34210 | IIc | - | - |
| ***WRKY118*** | Gm14:9976951..9978258 | Glyma14g11440 | IIc | - | - |
| ***WRKY119*** | Gm06:4091290..4091759 | Glyma06g05720 incorrect | IIc | Gene model short both ends | Gene model short at both ends |
| ***WRKY120*** | Gm08:1472858..1474401 | Glyma08g02160 | IIe | - | - |
| ***WRKY121*** | Gm05:40997970..40999444 | Glyma05g37390 | IIe | - | - |
| ***WRKY122*** | Gm05:36943122..36944876 | Glyma05g31910 | IIc | - | - |
| ***WRKY123*** | Gm06:16715202..16720290 | Glyma06g20300 | IIb | - | - |
| ***WRKY124*** | Gm02:1814827..1817115 | Glyma02g02430 | IIb | - | - |
| ***WRKY125*** | Gm01:4689528..4692149 | Glyma01g05050 | IIb | - | - |
| ***WRKY126*** | Gm04:40321797..40324993 | Glyma04g34220 incorrect | IIb | Gene model misses ATG | Gene model misses ATG |
| ***WRKY127*** | Gm08:5950312..5951589 | Glyma08g08290 incorrect | IIb | Genomic region is lacking some sequence | Genomic region is lacking some sequence |
| ***WRKY128*** | Gm08:6221601..6223386 | Glyma08g08720 | IIc | - | - |
| ***WRKY129*** | Gm05:31405708..31408531 | Glyma05g25270 incorrect | IIb | Gene model mis-predicts QVQR | Gene model mis-predicts QVQR |
| ***WRKY130*** | Gm05:31804493..31806863 | Glyma05g25770 | IIc | - | - |
| ***WRKY131*** | Gm05:812250..816075 | Glyma05g01280 | IIb | - | - |
| ***WRKY132*** | Gm11:1499870..1500966 | Glyma11g02361 | IIe | C-terminus non-canonical | C-terminus non-canonical |
| ***WRKY133*** | Gm09:9215912..9220906 | Glyma09g09400 | IIb | Gene model may be short at N-terminus | Gene model may be short at N-terminus |
| ***WRKY134*** | Gm12:8241106..8244216 | Glyma12g10350 | IIb | - | - |
| ***WRKY135*** | Gm04:4349614..4350727 | Glyma04g05700 | IIc | - | - |
| ***WRKY136*** | Gm04:4944596..4945852 | Glyma04g06480 incorrect | IIa | Gene model misses intron and N-terminus | Gene model misses intron and N-terminus |
| ***WRKY137*** | Gm04:4936179..4937258 | Glyma04g06470 | IIa | Gene model and FGENESH disagree on C-terminus | Gene model and FGENESH disagree on C-terminus |
| ***WRKY138*** | Gm14:10543971..10545687 | Glyma14g11960 | IIa | - | - |
| ***WRKY139*** | Gm09:29733003..29734454 | Glyma09g24080 | IIe | - | - |
| ***WRKY140*** | Gm08:43094949..43098475 | Glyma08g43260 incorrect | IIb | Gene model lacks N-terminal half | Gene model lacks N-terminal half |
| ***WRKY141*** | Gm07:12609707..12617323 | Glyma07g13610 incorrect | IIc | - | - |
| ***WRKY142*** | Gm03:33037360..33045449 | Glyma03g25770 | IIc | - | - |
| ***WRKY143*** | Gm16:2995485..2997474 | Glyma16g03570 | IIe | - | - |
| ***WRKY144*** | Gm16:33312638..33313276 | Glyma16g29500 incorrect | IIe | Glyma16g29560 is almost direct duplication | Glyma16g29560 is almost direct duplication |
| ***WRKY145*** | Gm04:47554440..47556577 | Glyma04g41700 incorrect | III | Gene model missed most of the gene | Gene model missed most of the gene |
| ***WRKY146*** | Gm06:14040836..14041778 | Glyma06g17690 incorrect | IIc | Gene model missed 5 and 3 prime ends | Gene model missed 5 and 3 prime ends |
| ***WRKY147*** | Gm15:19180387..19185976 | Glyma15g20990 | IIb | - | - |
| ***WRKY148*** | Gm13:35876809..35877192 | Glyma13g34250 incorrect | III | Gene model missed most of the gene | Gene model missed most of the gene |
| ***WRKY149*** | Gm06:10228675..10230804 | Glyma06g13090 | III | - | - |
| ***WRKY150*** | Gm16:2926836..2929341 | Glyma16g03480 | IIc | - | - |
| ***WRKY151*** | Gm06:12006135..12009585 | Glyma06g15260 | IIc | Gene model may be short at both ends | Gene model may be short at both ends |
| ***WRKY152*** | Gm10:39908336..39908517 | Glyma10g31410 incorrect | I | Gene model missed most of the gene | Gene model missed most of the gene |
| ***WRKY153*** | Gm10:39917896..39919687 | Glyma10g31420 incorrect | I | Gene model incorrect in WRKY domain | Gene model incorrect in WRKY domain |
| ***WRKY154*** | Gm15:11291206..11293979 | Glyma15g14860 | IIc | - | - |
| ***WRKY155*** | Gm18:48400269..48403724 | Glyma18g39970 | ? | - | - |
| ***WRKY156*** | Gm09:2438963..2441989 | Glyma09g03450 | IIe | - | - |
| ***WRKY181*** | Gm15:10844374..10847548 | Glyma15g14370 incorrect | IIe | Gene model misses N-terminus | Gene model misses N-terminus |
| ***WRKY158*** | Gm06:39491006..39494203 | Glyma06g37100 incorrect | I | Gene model misses N-terminus | Gene model misses N-terminus |
| ***WRKY159*** | Gm20:38962418..38963679 | Glyma20g30290 | IIe | - | - |
| ***WRKY160*** | Gm04:46251830..46253072 | Glyma04g40120 incorrect | III | Gene model misses N-terminal half of protein | Gene model misses N-terminal half of protein |
| ***WRKY161*** | Gm01:54210533..54211386 | Glyma01g43130 | IIe | Domain is longer than ususal | Domain is longer than ususal |
| ***WRKY162*** | Gm19:49828091..49830712 | Glyma19g44380 | III | - | - |
| ***WRKY163*** | Gm16:37210188..37212277 | Glyma16g34590 | III | - | - |
| ***WRKY164*** | Gm02:50284243..50285853 | Glyma02g46280 incorrect | IIb | Gene model misses N-terminal end | Gene model misses N-terminal end |
| ***WRKY165*** | Gm06:11541170..11542778 | Glyma06g14730 incorrect | III | Gene model misses most of gene | Gene model misses most of gene |
| ***WRKY166*** | Gm13:35883203..35884273 | Glyma13g34260 incorrect | III | Gene model misses most of gene | Gene model misses most of gene |
| ***WRKY167*** | Gm13:35894943..35896520 | Glyma13g34280 incorrect | III | Gene model misses most of gene | Gene model misses most of gene |
| ***WRKY168*** | Gm13:35869377..35870797 | Glyma13g34240 incorrect | III | Gene model misses N-terminus | Gene model misses N-terminus |
| ***WRKY169*** | Gm14:47189372..47190519 | Glyma14g37960 | ? | WRKY domain is short | WRKY domain is short |
| ***WRKY170*** | Gm09:44201245..44203320 | Glyma09g39040 | IIe | - | - |
| ***WRKY171*** | Gm14:45717567..45721055 | Glyma14g36430 | III | Glyma14g36430 and Glyma14g36440 similar repeat | Glyma14g36430 and Glyma14g36440 similar repeat |
| ***WRKY172*** | Gm14:45743592..45744827 | Glyma14g36446 | III | - | - |
| ***WRKY173*** | Gm14:45729563..45731082 | Glyma14g36438 | III | - | - |
| ***WRKY174*** | Gm03:226058..227858 | Glyma03g00460 | III | - | - |
| ***WRKY175*** | Gm17:31439928..31442914 | Glyma17g29190 | IId | - | - |
| ***WRKY176*** | Gm05:35354593,35374592 | Glyma05g29921 | - | Putative TIR-NBS-LRR-WRKY with kinase in intron | Putative TIR-NBS-LRR-WRKY with kinase in intron |
| ***WRKY177*** | Gm08:5992371..5996303 | Glyma08g08340 | ? | - | - |
|  |  |  |  |  |  |
|  |  |  |  |  |  |
|  |  |  |  |  |  |
|  |  |  |  |  |  |
|  |  |  |  |  |  |
|  |  |  |  |  |  |
|  |  |  |  |  |  |
|  |  |  |  |  |  |
|  |  |  |  |  |  |
|  |  |  |  |  |  |
|  |  |  |  |  |  |
|  |  |  |  |  |  |
|  |  |  |  |  |  |
|  |  |  |  |  |  |
| **Probable pseudogenes** |  |  |  |  |  |
|  |  |  |  |  |  |
| ***WRKYpg1*** | Gm01:43400758..43405757 | - | - | In frame stops. Next to transposon-like sequence | In frame stops. Next to transposon-like sequence |
| ***WRKYpg2*** | Gm12:33436284..33441283 | Glyma12g29970 | - | Missing C-terminal part. In frame stop and GAG poly. | Missing C-terminal part. In frame stop and GAG poly. |
| ***WRKYpg3*** | Gm06:28589287..28599286 | - | - | In frame stops. Next to transposon-like sequence | In frame stops. Next to transposon-like sequence |
| ***WRKYpg4*** | Gm14:45750065..45770064 | - | - | WRKY missing, GAG poly. Next to WRKY170, 171,172 | WRKY missing, GAG poly. Next to WRKY170, 171,172 |
| ***WRKYpg5*** | Gm05:22316949..22326948 | - | - | WRKY missing, GAG poly. | WRKY missing, GAG poly. |
| ***WRKYpg6*** | Gm15:18509437..18519436 | - | - | WRKY missing, CACTA transposon. | WRKY missing, CACTA transposon. |
| ***WRKYpg7*** | Gm20:44350390..44360389 | - | - | C-terminal part of domain missing. No transposon. | C-terminal part of domain missing. No transposon. |
| ***WRKYpg8*** | Gm15:46738419..46748418 | - | - | C-terminal part of domain missing. Transposon. | C-terminal part of domain missing. Transposon. |
| ***WRKYpg9*** | Gm01:6267916,6277915 | - | - | No WRKY part | No WRKY part |
| ***WRKYpg10*** | Gm08:6005538,6015537 | Glyma08g08370 | - | No WRKY part | No WRKY part |
| ***WRKYpg11*** | Gm17:26744603,26754602 | - | - | No WRKY part | No WRKY part |
| ***WRKYpg12*** | Gm05:41581323,41591322 | Glyma05g38200 | - | No WRKY part. In frame stop. | No WRKY part. In frame stop. |
| ***WRKYpg13*** | Gm19:34108703,34113702 | - | - | C-terminal part of domain missing. Transposon. | C-terminal part of domain missing. Transposon. |
| ***WRKYpg14*** | Gm05:16280053,16290052 | - | - | WRKY missing. Transposon. | WRKY missing. Transposon. |
| ***WRKYpg15*** | Gm16:24249352,24259351 | - | - | No WRKY part | No WRKY part |
| ***WRKYpg16*** | Gm12:35418204..35419106 | Glyma12g31860 | - | No WRKY part | No WRKY part |
| ***WRKYpg17*** | Gm09:28735313..28736138 | Glyma09g23270 | - | C-terminal part of domain missing. | C-terminal part of domain missing. |
| ***WRKYpg18*** | Gm06:45172914..45174125 | Glyma06g41910 | - | In frame stops in WRKY | In frame stops in WRKY |
| ***WRKYpg19*** | Gm12:22393464..22393609 | Glyma12g20900 | - | WRKY missing. Transposon. | WRKY missing. Transposon. |
| ***WRKYpg20*** | Gm05:41586227..41586703 | Glyma05g38200 | - | In frame stops missing WRKY | In frame stops missing WRKY |
| ***WRKYpg21*** | Gm09:14588320,14589319 | - | - | WRKY missing. Transposon. | WRKY missing. Transposon. |
| ***WRKYpg22*** | Gm20:32888766,32890765 | - | - | No WRKY part | No WRKY part |
| ***WRKYpg23*** | Gm17:25313159..25313933 | Glyma17g24700 | - | One domain missing large parts | One domain missing large parts |
| ***WRKYpg24*** | Gm19:33166744,33171743 | - | - | WRKY missing. Transposon. | WRKY missing. Transposon. |
| ***WRKYpg25*** | Gm07:20918593..20919466 | Glyma07g20510 | - | Missing 5 prime. Retrotransposon at 3 prime end. | Missing 5 prime. Retrotransposon at 3 prime end. |
| ***WRKYpg26*** | - | - | - | Impossible to find. Lots of 100% hits. | Impossible to find. Lots of 100% hits. |
| ***WRKYpg27*** | Gm14:43937946,43942945 | Glyma14g35150 | - | WRKY missing. Transposon. | WRKY missing. Transposon. |
| ***WRKYpg28*** | Gm15:32213942,32223941 | - | - | C-terminal part of domain missing. In frame stops. | C-terminal part of domain missing. In frame stops. |
| ***WRKYpg29*** | Gm20:22047658..22048200 | Glyma20g16010 | - | Probable pseudogene as GYN*QKYK for GYNWRKY in second domain | Probable pseudogene as GYN*QKYK for GYNWRKY in second domain |
| ***WRKYpg30*** | Gm14:11002636..11004011 | Glyma14g12290 | - | First domain in frame stop and frame shift | First domain in frame stop and frame shift |
| ***WRKYpg31*** | Gm03:32340121,32360120 | - | - | Possible pseudogene. Missing N-terminal part | Possible pseudogene. Missing N-terminal part |
| ***WRKYpg32*** | Gm10:15478720..15479844 | Glyma10g13720 |  | Gag-protease-integrase-RT-RNaseH downstream | Gag-protease-integrase-RT-RNaseH downstream |
| ***WRKYpg33*** | Gm15:19903373..19903444 | Glyma15g21570 | - | Incomplete WRKY domain. | Incomplete WRKY domain. |
| ***WRKYpg34*** | Gm15:42731483..42732790 | Glyma15g37120 | - | Flanked by CACTA transposon sequences | Flanked by CACTA transposon sequences |
| ***WRKYpg35*** | Gm17:25794237..25795375 | Glyma17g25150 | - |  |  |
| ***WRKYpg36*** | Gm17:25787689..25788628 | Glyma17g25140 | - | Both domains seem non functional was WRKY118 | Both domains seem non functional was WRKY118 |
| ***WRKYpg37*** | Gm15:42731483..42732790 | Glyma15g37120 | - | Flanked by CACTA transposon sequences | Flanked by CACTA transposon sequences |
| ***WRKYpg38*** | Gm05:41586227..41586703 | Glyma05g38200 | - | In frame stop. Missing WRKY. | In frame stop. Missing WRKY. |
| ***WRKYpg39*** | Gm08:28989837..28990680 | Glyma08g32740 | - | Gag-protease-integrase-RT-RNaseH downstream | Gag-protease-integrase-RT-RNaseH downstream |
| ***WRKYpg40*** | Gm17:39753107..39754816 | Glyma17g35750 | - | Retrotransposon protein at 3 prime end | Retrotransposon protein at 3 prime end |
| ***WRKYpg41*** | Gm10:16770304..16772465 | Glyma10g14610 | - | Flanked by retrotransposon Missing second H. | Flanked by retrotransposon Missing second H. |
| ***WRKYpg42*** | Gm13:6074457..6074710 | Glyma13g05720 | - | No WRKY part | No WRKY part |
