# Supplementary material for: Tobacco drought stress responses reveal new targets for Solanaceae crop improvement
Source: BMC Genomics. 2015 Jun 30;16(1):484. doi: 10.1186/s12864-015-1575-4 (PMC4485875; doi:10.1186/s12864-015-1575-4)
Supplement: Additional file 10: Figure S4. — Protein architecture of selected Group IX AP2/ERF transcription factors as determined by MEME. [file 12864_2015_1575_MOESM10_ESM.pdf]

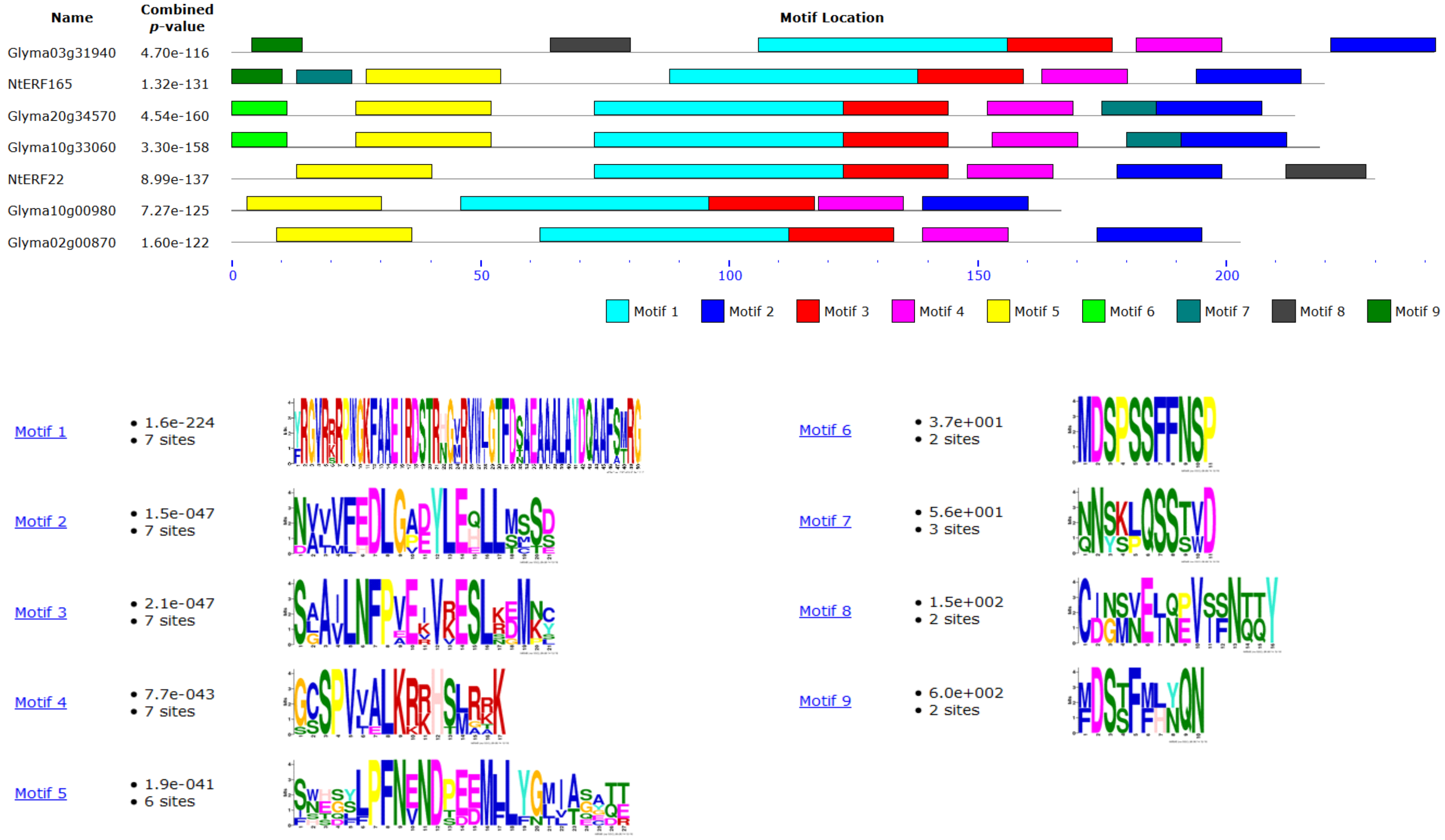
Name
Combined p-value
Motif Location
Glyma03g31940 4.70e-116
NtERF165 1.32e-131
Glyma20g34570 4.54e-160
Glyma10g33060 3.30e-158
NtERF22 8.99e-137
Glyma10g00980 7.27e-125
Glyma02g00870 1.60e-122
0
50
100
150
200
Motif 1
Motif 2
Motif 3
Motif 4
Motif 5
Motif 6
Motif 7
Motif 8
Motif 9
Motif 1 • 1.6e-224 • 7 sites
Motif 2 • 1.5e-047 • 7 sites
Motif 3 • 2.1e-047 • 7 sites
Motif 4 • 7.7e-043 • 7 sites
Motif 5 • 1.9e-041 • 6 sites
Motif 6 • 3.7e+001 • 2 sites
Motif 7 • 5.6e+001 • 3 sites
Motif 8 • 1.5e+002 • 2 sites
Motif 9 • 6.0e+002 • 2 sites
